# Graphene Quantum Dot Oxidation Governs Noncovalent Biopolymer Adsorption

**DOI:** 10.1038/s41598-020-63769-z

**Published:** 2020-04-27

**Authors:** Sanghwa Jeong, Rebecca L. Pinals, Bhushan Dharmadhikari, Hayong Song, Ankarao Kalluri, Debika Debnath, Qi Wu, Moon-Ho Ham, Prabir Patra, Markita P. Landry

**Affiliations:** 1 Department of Chemical and Biomolecular Engineering, University of California, Berkeley, Berkeley, CA 94720 USA; 2 Department of Biomedical Engineering, University of Bridgeport, Bridgeport, CT 06604 USA; 3 School of Materials Science and Engineering, Gwangju Institute of Science and Technology, Gwangju, 61005 South Korea; 4 Department of Mechanical Engineering, University of Bridgeport, Bridgeport, CT 06604 USA; 5 Innovative Genomics Institute (IGI), Berkeley, CA 94720 USA; 6 California Institute for Quantitative Biosciences, QB3, University of California, Berkeley, Berkeley, CA 94720 USA; 7 Chan-Zuckerberg Biohub, San Francisco, CA 94158 USA; 8 Department of Electrical and Computer Engineering & Technology, Minnesota State University, Mankato, MA 56001 USA

**Keywords:** Optical properties and devices, Organizing materials with DNA

## Abstract

Graphene quantum dots (GQDs) are an allotrope of carbon with a planar surface amenable to functionalization and nanoscale dimensions that confer photoluminescence. Collectively, these properties render GQDs an advantageous platform for nanobiotechnology applications, including optical biosensing and delivery. Towards this end, noncovalent functionalization offers a route to reversibly modify and preserve the pristine GQD substrate, however, a clear paradigm has yet to be realized. Herein, we demonstrate the feasibility of noncovalent polymer adsorption to GQD surfaces, with a specific focus on single-stranded DNA (ssDNA). We study how GQD oxidation level affects the propensity for polymer adsorption by synthesizing and characterizing four types of GQD substrates ranging ~60-fold in oxidation level, then investigating noncovalent polymer association to these substrates. Adsorption of ssDNA quenches intrinsic GQD fluorescence by 31.5% for low-oxidation GQDs and enables aqueous dispersion of otherwise insoluble no-oxidation GQDs. ssDNA-GQD complexation is confirmed by atomic force microscopy, by inducing ssDNA desorption, and with molecular dynamics simulations. ssDNA is determined to adsorb strongly to no-oxidation GQDs, weakly to low-oxidation GQDs, and not at all for heavily oxidized GQDs. Finally, we reveal the generality of the adsorption platform and assess how the GQD system is tunable by modifying polymer sequence and type.

## Introduction

Graphene is a two-dimensional hexagonal carbon lattice that possesses a host of unique properties, including exceptional electronic conductivity, mechanical strength, and adsorptive capacity^1–3^. However, graphene is a zero-bandgap material, and this lack of bandgap limits its use in semiconducting applications^4^. To engineer a bandgap, the lateral dimensions of graphene must be restricted to the nanoscale, resulting in spatially confined structures such as graphene quantum dots (GQDs)^5^. The bandgap of GQDs is attributed to quantum confinement^6,7^, edge effects^8^, and localized electron-hole pairs^9^. Accordingly, this gives rise to tunable fluorescence properties based upon GQD size, shape, and exogenous atomic composition. In comparison to conventional semiconductor quantum dots, GQDs are an inexpensive and less environmentally harmful alternative^10,11^. Moreover, for biological applications, GQDs are a low toxicity, biocompatible, and photostable material that offer a large surface-to-volume ratio for bioconjugation^11,12^.

Exploiting the distinct material properties of graphene often requires or benefits from exogenous functionalization. The predominant mechanism for graphene or graphene oxide (GO) functionalization is via covalent linkage to a polymer. However, noncovalent adsorption of polymers to carbon substrates is desirable in applications requiring reversibility for solution-based manipulation and tunable ligand exchange^13^, and preservation of the pristine atomic structure to maintain nanoscale graphene’s fluorescence characteristics^14^. Functionalization of graphene and GO has proven valuable for sensing and delivery applications. Optical sensors based on DNA-graphene or DNA-GO hybrids have been developed for the detection of nucleic acids^15,16^, proteins^17^, small molecules^18,19^, and metal ions^20^. Modifications to GO for drug delivery applications include PEGylation for higher biocompatibility^21,22^, covalent modification with functional groups for water solubility^23^, covalent linking of antibodies^24^, and noncovalent loading of anticancer drugs^21,23^. Noncovalent adsorption of polymers to graphene and GO has been predicted by theory and simulations^25,26^, and has occasionally been demonstrated experimentally^27^. In particular, single-stranded DNA (ssDNA) of varying lengths has been experimentally shown to noncovalently attach to graphene and GO, with hydrophobic and aromatic, π-π stacking electronic interactions posited to drive assembly^28,29^. Molecular dynamics (MD) simulations and density functional theory (DFT) modeling of these systems has enabled validation and mechanistic insight into the corresponding experimental findings^30,31^.

While noncovalent adsorption of DNA and various other polymers has been proposed by simulation and theory, and experimentally established as feasible for graphene and GO substrates, noncovalent polymer adsorption has not been fully investigated for their nanoscale counterparts: GQDs^32^. Noncovalent functionalization of GQDs with biopolymers offers the advantages of reversible binding and preserving the fluorescent substrate properties, while reducing graphene dimensions to the nanoscale enables two-dimensional carbon applications at the molecular scale, of relevance to study biological processes^33^. Herein, we present a facile protocol for noncovalent complexation of biopolymers to GQDs, with a focus on ssDNA. We explore the effects of GQD oxidative surface chemistry on the strength of binding interactions between surface-adsorbed polymers and GQDs, while preserving, or in some cases enabling, intrinsic GQD fluorescence. Ultimately, these results can serve as the basis for the design and optimization of polymer-GQD conjugates in various nanobiotechnology applications.

## Results

### GQD synthesis and characterization

**Figure 1:**
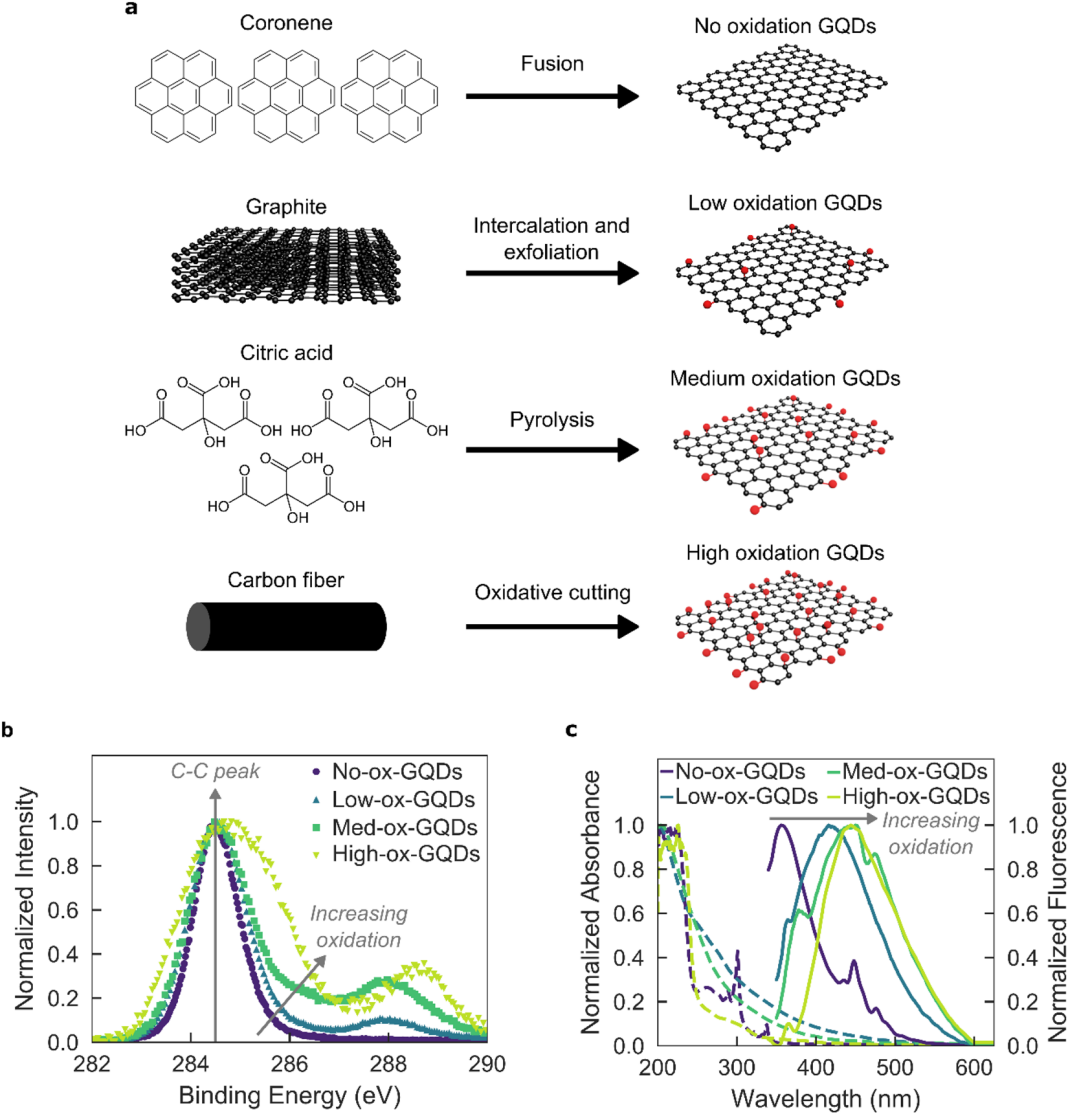
Four synthesis techniques are employed to produce graphene quantum dot (GQD) substrates of varying oxidation level. (**a**) Schematic illustration of synthesis techniques to produce no-oxidation GQDs (no-ox-GQDs), low-oxidation GQDs (low-ox-GQDs), medium-oxidation GQDs (med-ox-GQDs), and high-oxidation GQDs (high-ox-GQDs). **(b)** Normalized X-ray photoelectron spectroscopy (XPS) data of no-, low-, med-, and high-ox-GQDs. Arrows indicate the center of the C1s carbon-carbon (C-C) bond at 284.5 eV and increasing oxidation via contributions of various carbon-oxygen bonds (see Fig. S1 for deconvolutions and peak ratios). **(c)** Normalized absorbance (dashed) and fluorescence emission (solid) spectra of no-ox-GQDs in hexane solution and low-, med-, and high-ox-GQDs in water. All GQDs were excited at 320 nm.

We prepared and characterized four distinct GQD substrates of varying oxidation levels: no-oxidation GQDs (no-ox-GQDs) were fabricated by coronene condensation^34^, low-oxidation GQDs (low-ox-GQDs) by intercalation-based exfoliation^5^, medium-oxidation GQDs (med-ox-GQDs) by thermal decomposition of citric acid^35^, and high-oxidation GQDs (high-ox-GQDs) by carbon fiber cutting (Fig. 1a)^12^. X-ray photoelectron spectroscopy (XPS) was employed to quantify the differing oxidation levels among the GQD samples (Fig. 1b). The high-resolution carbon 1 s (C1s) XPS signal was deconvoluted into four individual peaks attributed to sp^2^ carbon-carbon bonds (284.5 eV), hydroxyl and epoxide groups (286.1 eV), carbonyl groups (287.5 eV), and carboxyl groups (288.7 eV) (Fig. S1)^36^. The peak area ratio of oxidized carbon (A_CO_) to sp^2^ carbon (A_CC_) decreases in the order of high-ox-GQDs (A_CO_/A_CC_ = 1.5) > med-ox-GQDs (0.45) > low-ox-GQDs (0.14) > no-ox-GQDs (0). Of note, no-ox-GQDs possessed only sp^2^ carbon, no oxidized carbon, in the C1s XPS spectrum. Atomic force microscopy (AFM) images of the GQDs revealed heights of high-ox-GQDs distributed between 0.5–3 nm, corresponding to 1–5 graphene layers, and heights of med- and low-ox-GQDs between 0.5-1 nm, indicating the presence of a single graphene layer (Fig. S2). The morphology of no-ox-GQDs was separately characterized by matrix-assisted laser desorption/ionization time-of-flight mass spectroscopy (MALDI-TOF MS) due to aggregation of no-ox-GQDs during AFM sample preparation hindering equivalent AFM analysis. The single graphene layer structure of no-ox-GQDs was determined by discrete peaks in the size distribution from MALDI-TOF MS, attributed to the presence of planar dimer, trimer, tetramer, pentamer, and hexamer fused-coronene structures (Fig. S3). Next, the fluorescence and absorbance spectra of low-, med-, and high-ox-GQDs were observed under 320 nm excitation in water (Figs. 1c and S4). The fluorescence maximum near 400 nm is described in previous literature as the intrinsic emission wavelength of GQDs with low oxidation, which is in close agreement with our own GQD samples^10^. GQD fluorescence peaks are observed at shorter wavelength as the GQD oxidation level decreases. As previously reported, longer wavelength emission emerges due to the presence of extrinsic, defect states^5,37^. No-ox-GQDs were insoluble in water due to the absence of oxygen-containing functional groups, and accordingly, aggregation led to self-quenched fluorescence. Instead, fluorescence of no-ox-GQDs was measured in hexane (Fig. 1c) and the fluorescence spectrum exhibits multiple peaks originating from the size distribution of no-ox-GQD multimers. The GQD excitation-emission profiles demonstrate that the optical characteristics of low-, med-, and high-ox-GQDs depend on the excitation wavelength, where the maximum fluorescence wavelength is red-shifted as the excitation is moved to longer wavelengths. However, the fluorescence of no-ox-GQDs does not show this spectral shift (Fig. S5). This excitation-wavelength dependence is commonly found in oxidized GQDs as a result of surface trap states introduced by functional groups and oxygen-related defects^38^. The no-ox-GQDs do not exhibit this excitation-dependency because they do not possess oxygen-containing functional groups.

### Noncovalent functionalization of GQDs with single-stranded DNA (ssDNA)

**Figure 2:**
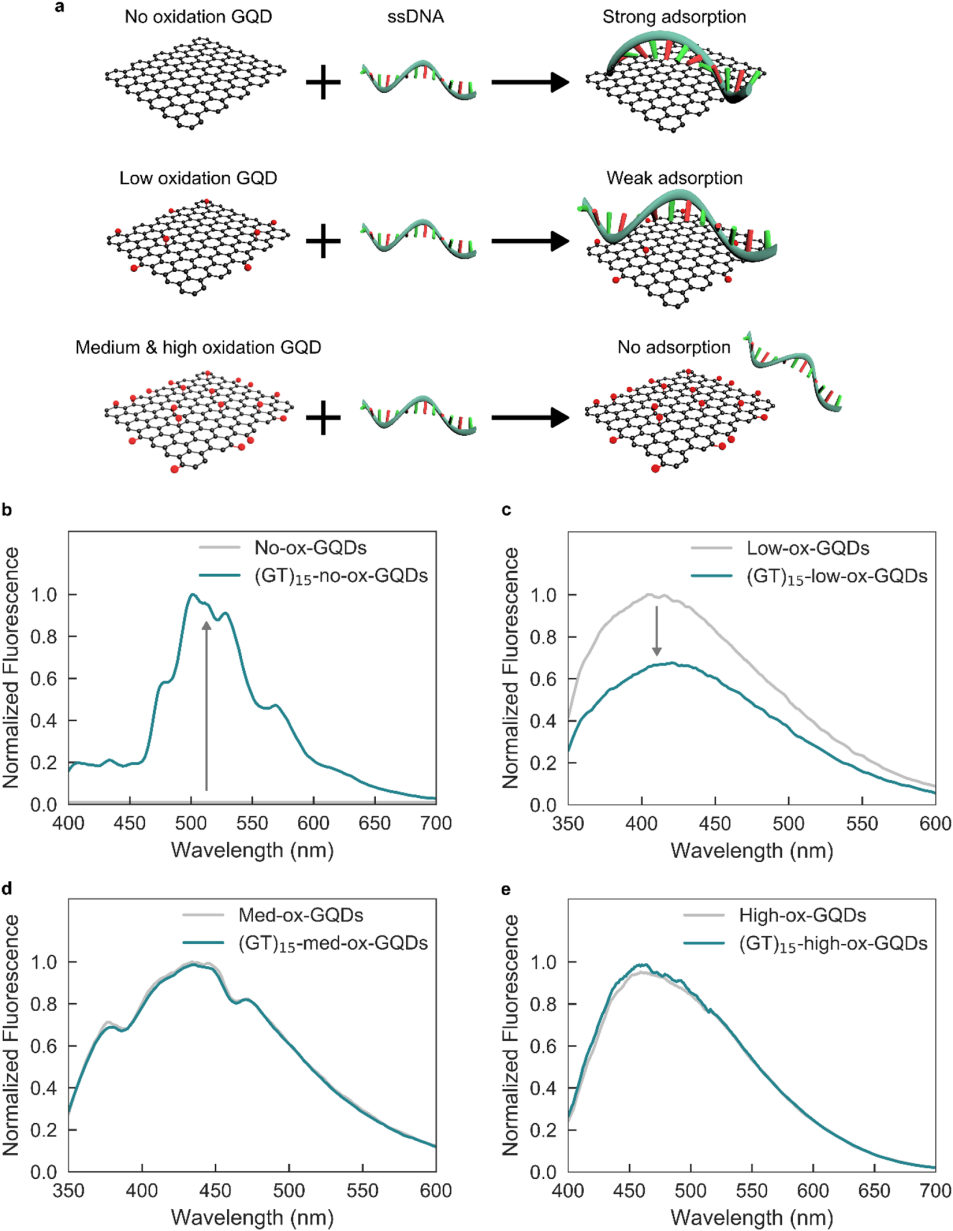
Single-stranded DNA (ssDNA)-GQD noncovalent interaction is governed by GQD oxidation level. **(a)** Schematic illustration of GQD oxidation level and resulting strength of ssDNA-GQD interaction. The noncovalent interaction between ssDNA and no-ox-GQDs is stronger than that of ssDNA and low-ox-GQDs. ssDNA does not adsorb to either med- or high-ox-GQDs. **(b–e)** Adsorption of (GT)_15_ ssDNA on the GQD surface results in GQD fluorescence modulation from before (gray) to after (blue) attempted adsorption of (GT)_15_ ssDNA for **(b)** no-ox-GQDs, **(c)** low-ox-GQDs, **(d)** med-ox-GQDs, and **(e)** high-ox-GQDs. The presence of ssDNA on the no-ox-GQDs is confirmed by an increase in fluorescence emission intensity from the initially insoluble no-ox-GQDs. The presence of ssDNA on the low-ox-GQDs results in a decrease in fluorescence intensity from the initially soluble low-ox-GQDs. No fluorescence intensity changes are observed for the med- and high-ox-GQDs, suggesting absence of ssDNA adsorption. Fluorescence spectra are normalized by the absorbance at 320 nm.

We next studied adsorption of the ssDNA sequence (GT)_15_ onto GQDs of varying oxidation levels (Fig. 2a). This ssDNA oligomer was chosen for initial adsorption studies based on its known π-π stacking adsorptive properties to the surface of pristine carbon nanotubes^39,40^, an analogous one-dimensional nanoscale substrate. For low-, med-, and high-ox-GQDs, ssDNA was added to GQDs in deionized (DI) water, the water was removed by vacuum evaporation to facilitate adsorption of (GT)_15_ onto the GQDs, then the GQD-ssDNA complexes were resuspended in water (mix-and-dry protocol). For no-ox-GQDs, an alternative complexation technique was employed because the as-synthesized no-ox-GQDs were insoluble in aqueous solution. Instead, the mixture of (GT)_15_ and solid no-ox-GQDs was probe-tip sonicated in phosphate-buffered saline (PBS) to disperse the hydrophobic GQD aggregates and enable ssDNA adsorption.

(GT)_15_ adsorption was verified by modulation of the intrinsic GQD fluorescence. For the initially soluble GQDs (low-, med-, and high-ox-GQDs), polymer adsorption manifests as fluorescence quenching from the original fluorescent state, whereas for the initially insoluble no-ox-GQDs, polymer adsorption manifests as fluorescence brightening from the original non-fluorescent, aggregated GQD state (Fig. 2). Fluorescence quenching was observed for low-ox-GQDs with (GT)_15_, but negligible fluorescence change was shown in the case of med- and high-ox-GQDs. These results suggest that (GT)_15_ does not adsorb to GQDs of higher oxidation levels. Additionally, increased quenching of low-ox-GQD fluorescence was demonstrated with a higher mass ratio of (GT)15 to low-ox-GQD (Fig. S6). Fluorescence quenching of low-ox-GQDs was not a result of Förster resonance energy transfer (FRET) because there is no spectral overlap between GQD emission and ssDNA absorption. (GT)_15_ adsorption also elicits a 15 nm red-shift of the low-ox-GQD fluorescence emission peak, resulting from either changing polarity proximal to the GQD surface or enrichment of larger GQDs (that display longer peak emission wavelengths) upon ssDNA adsorption (Fig. S7). This bathochromic shift is consistent for all biopolymers interacting with low-ox-GQDs. Interestingly, the simple mixing of (GT)_15_ with GQDs in the absence of drying results in only marginal fluorescence quenching for low-ox-GQDs (Fig. S8) and was accordingly ineffective in promoting ssDNA adsorption to GQDs. We hypothesize that the dehydration step is required to overcome electrostatic repulsion present in solution and enable close approach of the negatively charged ssDNA to the negatively charged oxidized GQDs. Moreover, water molecules solvating the low-ox-GQD surface may hinder initial contact of ssDNA with low-ox-GQDs^41^. We also investigated the effect of NaCl salt on ssDNA-GQD adsorption. For large GO, high salt concentration enhances ssDNA adsorption due to screening of repulsive electrostatic interactions between negatively charged GO and ssDNA, and among surface-adsorbed ssDNA^42^. However, NaCl does not facilitate adsorption of ssDNA onto high-ox-GQDs, and seems to disrupt adsorption onto low-ox-GQDs (Fig. S9). We hypothesize that the repulsive interactions between negatively charged GQDs and ssDNA is lessened due to the lower prevalence of oxidative functional groups in comparison to conventional GO. Moreover, repulsion among adsorbed ssDNA is less relevant for GQDs due to the smaller lateral dimensions as compared to GO sheets, resulting in fewer ssDNA molecules per GQD.

The (GT)_15_-no-ox-GQDs show a fluorescence increase from the initially non-fluorescent no-ox-GQD aggregates in aqueous solution and display the multiple absorption and emission peaks characteristic of the hexane-solubilized no-ox-GQDs (Fig. 1c). Thus, probe-tip sonication of no-ox-GQDs with (GT)_15_ was successful in dispersing no-ox-GQDs in PBS buffer by disrupting GQD aggregates and enabling the amphiphilic ssDNA to adsorb onto the exposed hydrophobic GQD surface, conferring water solubility to the complex. Without ssDNA, probe-tip sonication of no-ox-GQDs in solution does not result in a stable colloidal suspension. Presence of the ssDNA on no-ox-GQDs enabled AFM analysis of no-ox-GQDs and revealed heights distributed between 0.3-0.7 nm, corresponding to single graphene layer morphology (Fig. S3).

### Characterization of surface-bound ssDNA on GQD

**Figure 3:**
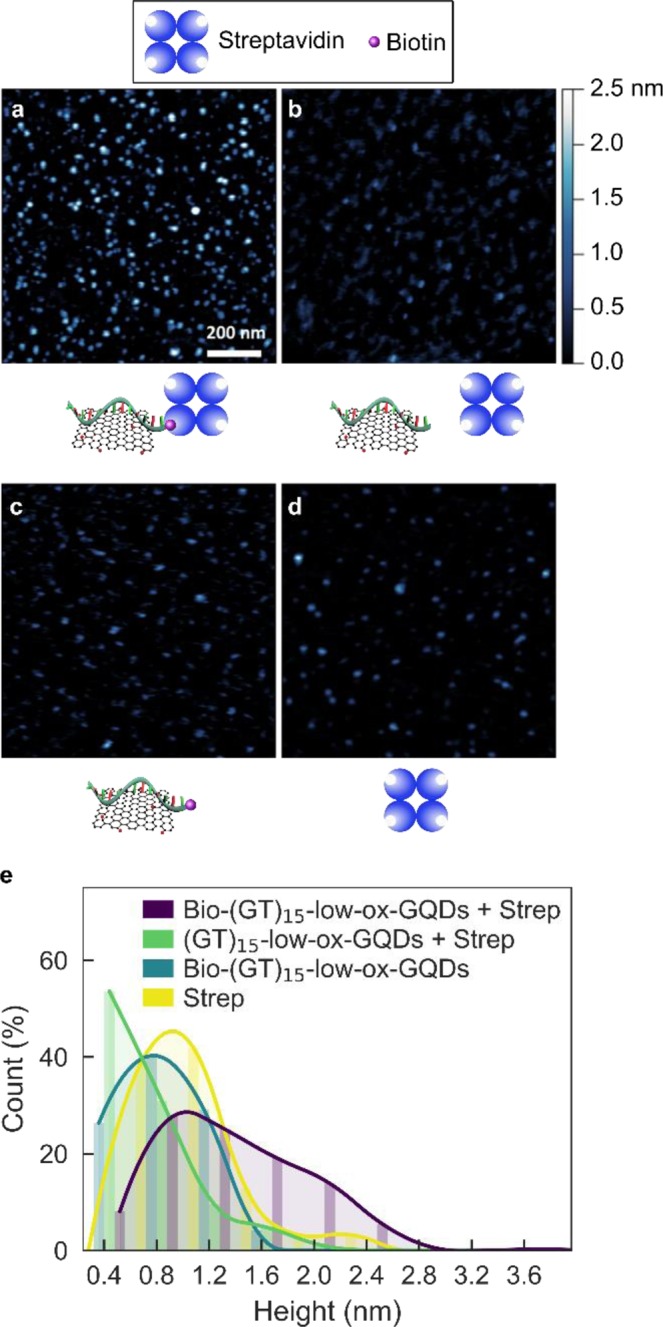
ssDNA adsorption to low-ox-GQDs is verified by atomic force microscopy (AFM). AFM images and accompanying schematics for **(a)** biotinylated-(GT)_15_-low-ox-GQDs and streptavidin (Bio-(GT)_15_-low-ox-GQD + Strep), **(b)** (GT)_15_-low-ox-GQDs and streptavidin ((GT)_15_-low-ox-GQD + Strep), **(c)** biotinylated-(GT)_15_-low-ox-GQDs (Bio-(GT)_15_-low-ox-GQD), and **(d)** streptavidin (Strep). Significantly larger heights in (A) are likely due to biotin-streptavidin binding via the biotinylated-ssDNA, which is adsorbed to the low-ox-GQD surface, absent in (**b**–**d**). **(e)** Corresponding height distribution histograms. Bin width is 0.4 nm and curve fits are added to guide the eye.

To verify the presence of ssDNA on low-ox-GQDs, we conducted AFM studies utilizing the well-known biotin-streptavidin interaction to impart a measurable change in the ssDNA-GQD height profile. This assay was required because the change in height due to ssDNA adsorption alone on the GQD surface is below the limit of detection by AFM. Biotin (or vitamin H) is a small molecule with a specific and strong binding affinity for the protein streptavidin (K_d_~10^−14^ mol/L). Biotinylated-(GT)_15_ was adsorbed to low-ox-GQDs with the mix-and-dry procedure to form Bio-(GT)_15_-low-ox-GQDs. Streptavidin was then mixed with the Bio-(GT)_15_-low-ox-GQDs in a 1:1 ratio of biotin:streptavidin (Bio-(GT)_15_-low-ox-GQDs + Strep) and the height profile of the resulting complexes was examined by AFM. Control samples of streptavidin mixed with non-biotinylated-(GT)_15_-low-ox-GQDs ((GT)_15_-low-ox-GQDs + Strep), biotinylated-(GT)_15_-low-ox-GQD only (Bio-(GT)_15_-low-ox-GQDs), and streptavidin only (Strep) were prepared for AFM analysis. Large biotin-streptavidin structures were frequently observed in the AFM images of Bio-(GT)_15_-low-ox-GQDs + Strep, and rarely found in the (GT)_15_-low-ox-GQDs + Strep sample, suggesting selective binding of streptavidin to the Bio-(GT)_15_-low-ox-GQDs (Fig. 3). Height distribution analysis reveals the percentage of structures larger than 1.8 nm is 20.3 ± 7.3% (mean ± standard deviation) for Bio-(GT)_15_-low-ox-GQDs + Strep, compared to only 0.5 ± 0.7% for (GT)_15_-low-ox-GQDs + Strep, 0% for Bio-(GT)_15_-low-ox-GQDs, and 6.9 ± 5.0% for Strep. Here, the threshold value of 1.8 nm is the experimental sum of the GQD average height (0.6 nm) and streptavidin average height (1.2 nm). Accordingly, this confirms the formation of specific streptavidin-biotin-(GT)_15_-low-ox-GQD complexes, and thus suggests the successful noncovalent adsorption of ssDNA on the surface of low-ox-GQDs. Absence of (GT)_15_ adsorption onto med-ox-GQDs was also demonstrated with this assay by preparing a mixture of biotinylated-(GT)_15_ and med-ox-GQDs (Bio-(GT)_15_-med-ox-GQDs) by the same method and adding streptavidin (Bio-(GT)_15_-med-ox-GQDs + Strep). Height distribution analysis reveals the percentage of structures >1.8 nm is 9.9 ± 0.6% for Bio-(GT)_15_-med-ox-GQDs + Strep, close to the control value of 7.5 ± 2.6% obtained for non-specific adsorption of streptavidin onto med-ox-GQDs and (GT)_15_ lacking biotin (Fig. S10). This result, in corroboration with the lack of fluorescence quenching, verifies that ssDNA does not form stable adsorbed structures with med-ox-GQDs.

### Sequence-dependent adsorption of ssDNA onto the GQD surface

**Figure 4:**
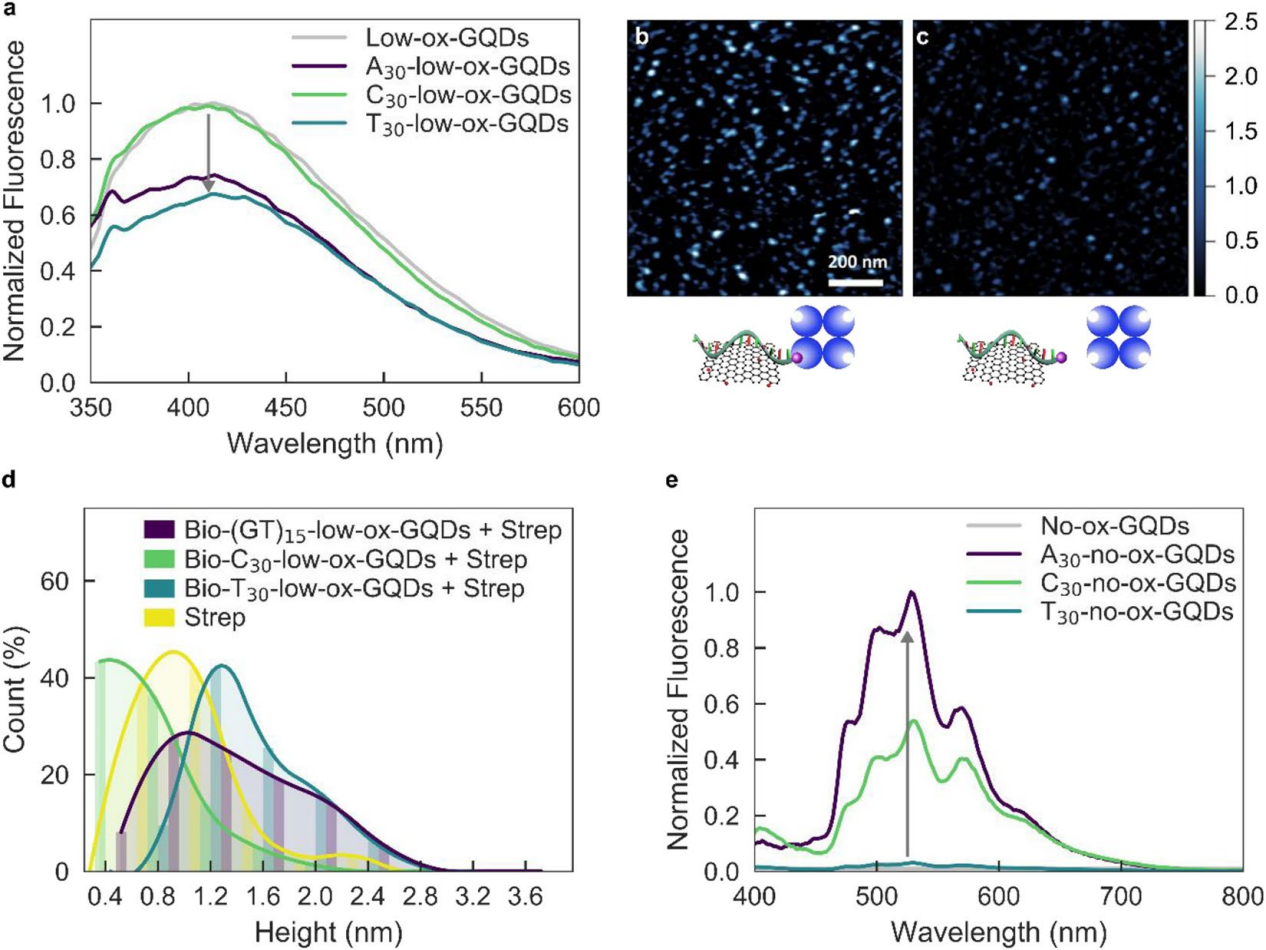
Propensity of ssDNA adsorption to low- and no-ox-GQDs depends on ssDNA sequence. (**a**) Fluorescence spectra of low-ox-GQDs (gray) and low-ox-GQDs with either A_30_, C_30_, or T_30_ adsorbed by the mix-and-dry process. AFM images and accompanying schematics for **(b)** biotinylated-T_30_-low-ox-GQDs and streptavidin (Bio-T_30_-GQD + Strep) and **(c)** biotinylated-C_30_-low-ox-GQDs and streptavidin (Bio-C_30_-GQD + Strep). **(d)** Corresponding height distribution histograms. Bin width is 0.4 nm and curve fits are added to guide the eye. **(e)** Fluorescence spectra of no-ox-GQDs (gray) and no-ox-GQDs with either A_30_, C_30_, or T_30_ adsorbed by probe-tip sonication. All GQD fluorescence spectra are normalized by the absorbance at 320 nm.

To examine the effect of ssDNA nucleotide sequence on GQD adsorption affinity, we investigated the adsorption affinities of three ssDNA oligomers of the same length but different nucleotide identities: poly-adenine, A_30_; poly-cytosine, C_30_; and poly-thymine, T_30_. Poly-guanine, G_20_, a shorter ssDNA oligomer than other ssDNA candidates, was used as the poly-G model case because of commercial unavailability of longer poly-G ssDNA oligomers. To adsorb ssDNA polymers to low-ox-GQDs, each A_30_, C_30_, G_20_ and T_30_ ssDNA oligomer was mixed and dried with low-ox-GQDs to form ssDNA-GQD complexes: A_30_-low-ox-GQDs, C_30_-low-ox-GQDs, G_20_-low-ox-GQDs, and T_30_-low-ox-GQDs. Following ssDNA adsorption, the integrated fluorescence intensity of low-ox-GQDs decreased to 76.1 ± 8.2% (mean ± standard deviation) for A_30_, 85.1 ± 1.9% for G_20_, 72.0 ± 6.9% for T_30_ on average, and maintained the original value for C_30_ (Figs. 4a and S11). These results suggest that A_30_, G_20_, and T_30_ adsorb onto the low-ox-GQD surface, while C_30_ does not adsorb. We repeated the AFM studies with low-ox-GQD substrates to which we adsorbed biotinylated-C_30_ and mixed this construct with streptavidin (Bio-C_30_-low-ox-GQDs + Strep) to further investigate whether C_30_ adsorbs to low-ox-GQDs. As a positive control for adsorption, biotinylated-T_30_ was prepared and incubated with streptavidin (Bio-T_30_-low-ox-GQDs + Strep). AFM imaging of the biotinylated-ssDNA, low-ox-GQD, and streptavidin mixture demonstrated that Bio-C_30_-low-ox-GQDs and Strep were observed as separate structures, while Bio-T_30_-low-ox-GQDs + Strep displayed heights consistent with the larger, assembled complex. Specifically, the height distribution analysis shows the percentage of structures>1.8 nm is 1.6 ± 0.4% for Bio-C_30_-low-ox-GQDs and Strep, which is significantly lower than the value of 12.6 ± 8.5% for Bio-T_30_-low-ox-GQDs + Strep (Fig. 4b–d). These results, consistent with our fluorescence quenching assay, suggest that C_30_ does not adsorb onto the low-ox-GQD surface.

To further understand the sequence dependence of ssDNA adsorption to GQD substrates, we studied ssDNA adsorption affinity of A_30_, C_30_, and T_30_ ssDNA oligomers to no-ox-GQDs. A_30_, C_30_, and T_30_ ssDNA oligomers were probe-tip sonicated with water-insoluble no-ox-GQDs. All three ssDNA sequences resulted in stable colloidal dispersions of ssDNA-coated no-ox-GQDs. The relative fluorescence intensities normalized by the absorbance at the excitation wavelength (320 nm) establishes the fluorescence quantum yield order as A_30_ > C_30_ > T_30_ (Fig. 4d). However, it is noteworthy that this order does not directly reflect the adsorption affinity of each polynucleotide, as the fluorescence intensity is correlated to both nucleotide-specific adsorption affinity and quenching ability. Yet, this result still implies higher adsorption proclivity for C_30_ on no-ox-GQDs over low-ox-GQDs.

### Strength of ssDNA-GQD interactions

Next, we investigated ssDNA desorption from ssDNA-coated low-ox-GQDs and no-ox-GQDs by using high temperature and complementary ssDNA (cDNA) methods. To study the effect of high temperature, ssDNA-GQD samples of A_30_, C_30_, and T_30_ on either no- or low-ox-GQDs were prepared. Fluorescence of each GQD sample was measured at room temperature before and after heating samples to 50 °C for 2 hours to attempt desorption of ssDNA from GQDs (Fig. S12a,b). As expected, no fluorescence change was observed after heating pristine low-ox-GQDs and C_30_-low-ox-GQDs because these samples did not initially have surface-adsorbed ssDNA. Fluorescence intensity of A_30_- and T_30_-low-ox-GQDs increased after heating, indicating that 47.4% of A_30_ and 30.7% of T_30_ desorbed from the low-ox-GQD surface upon heating to 50 °C. In comparison, all ssDNA-no-ox-GQDs maintained their initial fluorescence intensity after heating to 50 °C, suggesting this heat treatment was insufficient to desorb ssDNA from the pristine no-ox-GQD carbon lattice. When ssDNA-no-ox-GQDs were instead heated to 95 °C for 2 hours, fluorescence intensities of all groups significantly decreased, indicating that 41.9% of A_30_, 43.6% of C_30_, and 39.3% of T_30_ desorbed from the no-ox-GQD surface (Fig. S14). This difference in temperature stability implies that the adsorption affinity of ssDNA on the GQD surface is stronger for no-ox-GQDs than for low-ox-GQDs. A recent MD simulation study reported that the estimated binding free energy between T_20_ ssDNA and GO increased significantly when the oxygen content of GO was reduced to below 10%^30^. Accordingly, we hypothesize that GQD oxidation level is directly related to the adsorption affinity between ssDNA and GQDs. Stronger adsorption affinity of ssDNA on no-ox-GQDs results from increased sp^2^ graphitic carbon content available for π-π stacking interactions with ssDNA and reduced negative GQD surface charge for electrostatic repulsion.

Adsorption stability of ssDNA on GQDs was also studied with a hybridization assay, where ssDNA complementary to the adsorbed sequence, cDNA, hybridizes in solution phase with the GQD surface-adsorbed ssDNA. It is known that double-stranded DNA has a low adsorption affinity for GO surfaces, and this property has been previously used to study the adsorption affinity of ssDNA by cDNA-induced desorption^42^. The cDNA oligomer (AC)_15_ was added to either (GT)_15_-low-ox-GQD or (GT)_15_-no-ox-GQD solutions in five-fold excess relative to (GT)_15_. Resulting fluorescence profiles were measured 2 hours following addition of (AC)_15_ and compared with the initial fluorescence profile (Fig. S12c,d). Fluorescence of low-ox-GQDs decreased to 68% upon initial (GT)_15_ adsorption, then recovered to 88% of the initial low-ox-GQD fluorescence due to ssDNA desorption in the presence of cDNA. On the other hand, fluorescence of (GT)_15_-no-ox-GQDs maintained the initial fluorescence value after adding cDNA. The addition of non-complementary T_30_ ssDNA onto (GT)_15_-low-ox-GQDs did not induce the desorption of (GT)_15_ (Fig. S13). These results further substantiate our conclusion that ssDNA adsorbs to no-ox-GQDs more strongly than to low-ox-GQDs.

### Molecular dynamics simulation of ssDNA-GQD interactions

**Figure 5:**
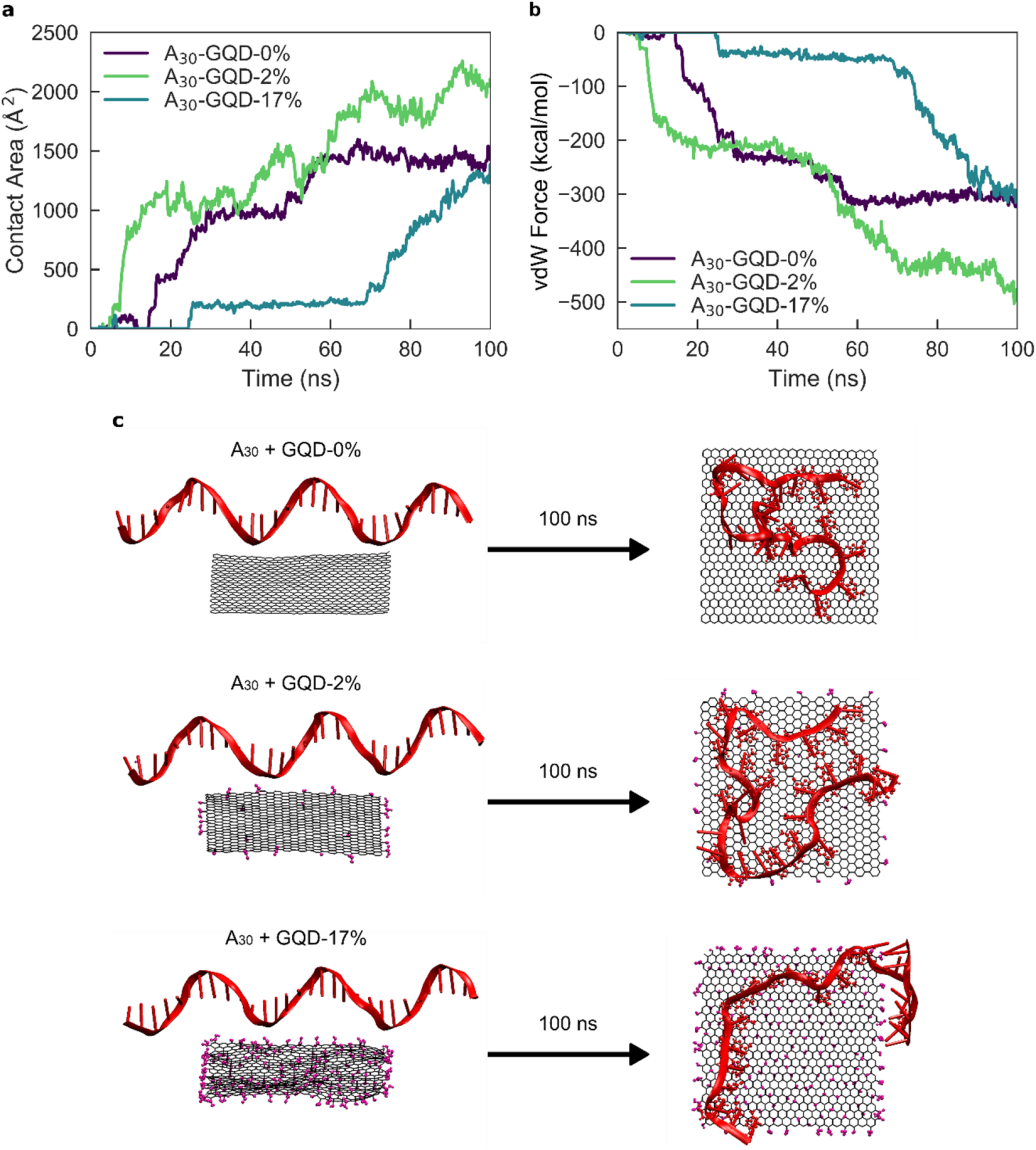
Molecular dynamics simulations confirm A_30_ ssDNA adsorption dependency on GQD oxidation level. Time-dependent **(a)** contact area and **(b)** van der Waals interactions for A_30_ ssDNA adsorbing to GQD-0%, GQD-2%, and GQD-17%. **(c)** Initial (left) and final (right) configurations of A_30_ ssDNA with GQD-0%, GQD-2%, and GQD-17% for a 100 ns simulation.

To understand the time-dependent energetics and structures of the ssDNA-GQD binding process, we performed MD simulations of ssDNA oligomers adsorbing to GQDs of varying oxidation levels. To investigate how GQD surface polarity affects ssDNA adsorption, we analyzed non-bonding interaction energies between A_30_ ssDNA and differentially oxidized GQDs during a 100 ns MD simulation. We utilized three types of GQDs, with 0%, 2.28%, and 17.36% oxidation (denoted as GQD-0%, GQD-2%, and GQD-17%, respectively), calculated as the ratio of oxidized carbon to sp^2^ carbon. Overall, our results indicate that ssDNA physisorption is driven by a combination of van der Waals’s (vdW) interactions and hydrogen bonding (H-bonding) to the GQD. Based on the contact area of ssDNA on the GQD surface, center-of-mass distance, and number of atoms within 5 Å of the GQD surface, A_30_ is more closely adsorbed on less oxidized GQD surfaces, such as GQD-0% and GQD-2%, as compared to the more highly oxidized surface of GQD-17% (Figs. 5a and S15a,b). These results indicate that vdW interactions are the sole contributor towards the adsorption of A_30_ on GQD-0%, whereas H-bonding marginally contributes to the adsorption of A_30_ on GQD-2% and GQD-17% in addition to dominant vdW interactions (Figs. 5b and S15c). These interactions further support the less significant role of hydrogen bonds between the ssDNA and oxygen groups on the GQDs. In the simulation for GQD-17%, A_30_ showed negligible contact with the GQD until 70 ns, in comparison with A_30_ contact within 20 ns for the less oxidized GQD cases. After 70 ns, transient contact of A_30_ with GQD-17% was observed, as signified by the fluctuating center-of-mass distances, the latter suggesting highly unstable physisorption of A_30_ on GQD-17%. These MD results suggest more stable adsorption of A_30_ onto less oxidized GQDs (GQD-0% and GQD-2%) as compared to GQD-17%, and agree with experimentally determined selective adsorption of ssDNA on no- and low-ox-GQDs, which is not observed in the case of med- and high-ox-GQDs.

We next investigated the dependency of ssDNA-GQD adsorption on nucleotide sequence by performing MD simulations of A_30_, C_30_, and T_30_ ssDNA onto GQD-0% and GQD-2% (Figs. S16 and S17). While A_30_ and T_30_ displayed similar adsorption dynamics onto GQD-0% and GQD-2%, C_30_ adsorbed more weakly onto GQD-0% and GQD-2%, in alignment with previous studies regarding the interaction of homopolynucleotides with graphite^31,32^. For the GQD-0% case, the simulation shows that only the 5′ end of C_30_ interacts with the GQD-0% surface, while the other end attempts self-hybridization and consequently unfolds. C_30_ does not show any attractive interaction with GQD-2%, corroborating experimental results that C_30_ does not quench low-ox-GQD fluorescence and was not found in appreciable quantities on the low-ox-GQD surface by AFM. Overall, the MD simulations recapitulate experimental findings that GQD oxidation level determines the ssDNA interaction with and conformation on the GQD surface, and that the ssDNA-GQD-2% interaction is strongly dependent on the nucleotide sequence.

### Platform extension to other biomolecule-GQD constructs

**Figure 6:**
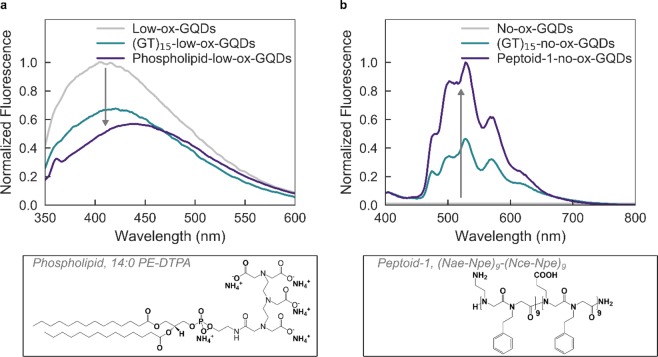
Noncovalent surface adsorption of biopolymers to low- and no-ox-GQDs is demonstrated by fluorescence modulation upon adsorption of phospholipid (14:0 PE-DTPA) and Peptoid-1 ((Nae–Npe)_9_-(Nce–Npe)_9_), respectively. (**a**) Normalized fluorescence emission spectra of low-ox-GQDs taken before (gray) and after (purple) the mix-and-dry process with phospholipid, 14:0 PE-DTPA. **(b)** Normalized fluorescence emission spectra of no-ox-GQDs taken before (gray) and after (purple) probe-tip sonication with Peptoid-1, (Nae-Npe)_9_-(Nce-Npe)_9_. The (GT)_15_-no-ox-GQDs spectrum (blue) is included for comparison. All GQD fluorescence spectra are normalized by the absorbance at 320 nm.

Finally, we demonstrate that this noncovalent adsorption platform is extendable to other biomolecules on GQDs. We hypothesized that planar sheet- or bilayer-forming molecules would be amenable for adsorption onto a two-dimensional GQD substrate^43^. Accordingly, we attempted and successfully created biopolymer-GQD constructs with two such structure-forming biomolecules, phospholipids and peptoids. The phospholipid, 1,2-distearoyl-sn-glycero-3-phosphoethanolamine-N-diethylenetriaminepentaacetic acid (14:0 PE-DTPA), was adsorbed onto low-ox-GQDs with the same mix-and-dry protocol as ssDNA, and resulted in the expected fluorescence quenching of low-ox-GQDs that confirms adsorption (Fig. 6a). As is the case with ssDNA adsorption, this fluorescence quenching may be due to a charge transfer mechanism between the GQD and adsorbed polymer^44^.

A peptoid is a synthetic peptide in which the variable group is attached to the amine rather than the alpha carbon, resulting in a loss of the chiral center^45^. In particular, 36-mer peptoids with alternating ionic and hydrophobic sidechains have been designed as amphiphilic, sheet-forming peptoids^46^. Two peptoid sequences were tested: Peptoid-1 is a diblock of alternating N-(2-aminoethyl) glycine (Nae) and N-(2-phenethyl) glycine (Npe) units, abbreviated (Nae–Npe)_9_, and N-(2-carboxyethyl) glycine (Nce) and Npe, abbreviated (Nce–Npe)_9_. Electrostatic interactions between the amine and carboxyl groups drive solution-phase self-assembly of these 36-mers into nanosheet morphology. Peptoid-2 is simply (Nce–Npe)_18_, with only the carboxyl sidechain present. Therefore, no amine-carboxyl ionic interactions are available to initiate assembly and this peptoid is incapable of forming stable nanosheets by itself. Probe-tip sonication of no-ox-GQDs with Peptoid-1, (Nae–Npe)_9_-(Nce–Npe)_9_, resulted in a stable Peptoid-1-no-ox-GQD suspension (Fig. 6b). The phenyl sidechains are posited to π-π stack with the no-ox-GQD basal plane in the same manner as ssDNA, thus resulting in stable constructs. Peptoid-2, (Nce–Npe)_18_, was not able to suspend the no-ox-GQDs, most likely due to the absence of electrostatic interactions between the peptoids required to form stable sheet nanostructures and presumably also a GQD surface coating.

## Discussion

We have demonstrated the feasibility of, and developed procedures for, noncovalent adsorption of ssDNA, phospholipids, and peptoid polymers to GQDs (summarized in Table S1). To the best of our knowledge, this is the first experimental realization of the noncovalent physisorption of these biomolecules on GQDs, which has not been previously investigated due to challenges arising from the small size and variable oxidation of GQD substrates^15^. We have confirmed the perturbative role of GQD oxidation on ssDNA adsorption, and further investigated the varying adsorption and desorption properties of ssDNA based on the GQD oxidation level and ssDNA sequence. To this end, four types of photoluminescent GQDs with different oxidation levels were synthesized. Characterization of the four GQD types reveals that changing GQD oxidation level allows tuning the GQD optical fingerprints. This finding presents opportunities to create libraries of GQDs displaying unique photoluminescent properties, or the ability to identify GQDs by means of fluorescence profiles. ssDNA adsorption to GQD substrates is assessed by photoluminescence modulation and morphological characterization. Specifically, adsorption of ssDNA on low-ox-GQDs is confirmed by fluorescence quenching and AFM studies, and the adsorption affinity is evaluated by high temperature ssDNA desorption and by hybridization. Adsorption of ssDNA on no-ox-GQDs is confirmed by producing stable colloidal suspensions with fluorescence emission, whereby higher ssDNA sequence adsorption affinity resists disruption by high temperature or cDNA. Thus, GQD oxygen content determines ssDNA adsorption affinity, where ssDNA can adsorb on no- and low-ox-GQD surfaces but not on med- nor high-ox-GQDs. This preferential ssDNA adsorption implies that ssDNA adsorbs more favorably onto graphene-like carbon domains rather than oxidized carbon domains^42^, underscoring the role of interfacial π-π electronic interactions between the GQD and aromatic ssDNA nitrogenous bases contributing more than hydrogen bonds between oxygen groups on the GQD with the ssDNA. Likewise, the surface roughness and electrostatic repulsion created by oxygen groups on the med- and high-ox-GQDs could prevent effective π-π stacking interactions of ssDNA nucleobases with the GQD graphitic surface, consequently inhibiting adsorption.

ssDNA attachment on low-ox-GQDs is sequence-dependent: poly-A, G, T do adsorb to low-ox-GQDs, while poly-C does not adsorb. Previously, Sowerby *et al*. reported adsorption affinities of the four DNA bases on graphite (as determined by column chromatography) in decreasing order of G > A > T > C^47^, in accordance with our results showing a low adsorption affinity of cytosine to GQDs. Likewise, for pyrimidine homopolymers studied with chemical force microscopy, T_50_ required a much stronger peeling force of 85.3 pN from graphite as compared 60.8 pN for C_50_^48^. Conversely, a recent study suggests that poly-C interacts with a carboxylated GO surface more strongly than poly-T or poly-A^49^. This result is attributed to the fact that poly-C ssDNA readily forms secondary structures, enabling hydrogen bonding interactions between the folded ssDNA and GO that drive the adsorption process^30^. However, our low-ox-GQDs contain significantly less oxidative functional groups available for hydrogen bonding (A_CO_/A_CC_ = 0.14) in comparison to common GO (A_CO_/A_CC_~0.36)^50^, therefore we conclude that C_30_ does not interact with the same binding modalities as shown with non-nanoscale GO. From our MD simulations, C_30_ again does not show any attractive interaction with GQD-2% and shows some adsorption to GQD-0%. Another recent study has discovered that unfolded poly-C, rather than the i-motif secondary structure, has higher affinity for graphene oxide surfaces^51^. Accordingly, we posit that π-π stacking interactions between the aromatic portions of ssDNA and pristine graphitic areas of GQDs can overcome intramolecular forces holding together the C_30_ secondary structure, resulting in some adsorption of unfolded C_30_ to GQD-0%. Thus, contact with pristine GQDs may prompt poly-C unfolding and result in selective adsorption, whereas oxidized GQDs may be unable to disrupt potential C_30_ secondary structures to support stable surface adsorption.

In sum, the effect of graphene-based substrate size on biomolecule adsorption for nanoscale GQDs in comparison to micro-/macroscale GO sheets is best established by considering (1) solution ionic strength and (2) biopolymer sequence dependency. Towards (1), introduction of salt precludes ssDNA adsorption onto low-ox-GQDs, yet not onto no-ox-GQDs, whereas salt is known to assist ssDNA adsorption onto GO. Towards (2), we find differing ssDNA sequence dependencies on GQDs in comparison to their GO counterparts, particularly for poly-C.

Applications of graphene-based nanomaterials are vast, and a better understanding of parameters that affect adsorption of polymers to GQDs are needed to enable future applications in diagnostics, biomolecule delivery, and sensing. Our noncovalent attachment protocols to synthesize ssDNA-GQD complexes can lead to new opportunities in developing GQD-based nucleic acid detection platforms and further biological molecular sensing and imaging applications. Moreover, we show the adsorption protocols developed for ssDNA are generic to adsorb other biopolymers, such as phospholipids and peptoids, to GQDs. Successful synthesis of the Peptoid-1-GQD construct motivates future developments in biopolymer-GQD-based detection platforms with peptoid-mediated protein recognition^52^. The noncovalent adsorption of biopolymers beyond ssDNA to GQDs provides a route to tune the GQD system not only by choice of GQD color and oxidation level, but additionally by polymer sequence and type. The platform developed here can be leveraged to expand the current possibilities of designing and applying GQD-based nanotechnologies.

## Methods

### Preparation of no-oxidation GQDs (no-ox-GQDs)

No-ox-GQDs were prepared according to previous literature^34^. Briefly, 20 mg of coronene (95%, Acros) was vacuum-sealed in a glass ampule and annealed at 500 °C for 20 hours. After cooling to room temperature, the product was loaded into a quartz tube and annealed at 500 °C for 30 min under H_2_ and Ar atmosphere (10 and 200 sccm, respectively) to remove unreacted coronene.

### Preparation of low-oxidation GQDs (low-ox-GQDs)

Low-ox-GQDs were prepared by an intercalation-based exfoliation method^5^. 20 mg of graphite powder (natural, briquetting grade, -100 mesh, 99.9995%, UCP-1 grade, Ultra “F” purity, Alfa Aesar) and 300 mg of potassium sodium tartrate tetrahydrate (>99%, Sigma-Aldrich) were ground in a mortar and pestle. The powder was transferred to a glass tube and heated in a tube furnace at 250 °C for 24 hours under Ar gas. The product powder was dispersed in 30 mL of deionized (DI) water and ultrasonicated for 10 min (Branson Ultrasonic 1800). The translucent, brown solution was centrifuged at 3220 g for 30 min and the supernatant was collected. For desalting and size selection, the solution was spin-filtered using a 100 kDa molecular weight cutoff (MWCO) centrifugal filter (Amicon Ultra-15, Ultracel, Millipore) at 3220 g for 30 min and the eluent solution was collected. The final product solution was spin-filtered with a 3 kDa centrifugal filter at 3220 g for 30 min to remove residual salts, repeated six times, and the remnant solution was collected.

### Preparation of medium-oxidation GQDs (med-ox-GQDs)

Med-ox-GQDs were prepared according to previous literature^35^. 2 g of citric acid (>99.5%, ACS reagent, Sigma-Aldrich) was added to a 20 mL vial and heated to 200 °C in a heating mantle for 30 min until citric acid liquified into an orange solution. The solution was cooled to room temperature and added dropwise into 100 mL of 10 mg/mL NaOH solution while stirring. The pH of the med-ox-GQDs solution was neutralized to pH 7 by adding NaOH. The final product solution was spin-filtered with a 3 kDa centrifugal filter at 3220 g for 30 min to remove residual salts, repeated six times, and the remnant solution was collected.

### Preparation of high-oxidation GQDs (high-ox-GQDs)

High-ox-GQDs were prepared according to previous literature^12^. Briefly, 0.3 g of carbon fibers (>95%, carbon fiber veil, Fibre Glast) was added into a mixture of concentrated H_2_SO_4_ (60 mL) and HNO_3_ (20 mL). The mixture was ultrasonicated for two hours and stirred for 24 hours at 120 °C. The solution was cooled to room temperature and diluted with DI water (800 mL). The pH of the high-ox-GQDs solution was adjusted to pH 8 by adding Na_2_CO_3_. The final product solution was spin-filtered with a 3 kDa centrifugal filter at 3220 g for 30 min to remove residual salts, repeated six times, and the remnant solution was collected.

### Fabrication of ssDNA-GQD complex by mix-and-dry process

10 µg of GQDs were mixed with 10 nmol of ssDNA dissolved in 0.2 mL DI water. The mixture was dried for 4 hours in a 1.5 mL microcentrifuge tube under moderate vacuum (~5 torr). The dried solid was re-dispersed in 1 mL DI water.

### Fabrication of ssDNA-no-ox-GQD complex by probe-tip sonication process

1 mg of no-ox-GQDs and 100 nmol of ssDNA was dispersed in 1 mL PBS buffer at pH 7.4. The mixture was ultrasonicated for 2 min and probe-tip sonicated for 30 min at 5 W power in an ice bath (Cole Parmer Ultrasonic Processor, 3 mm tip diameter). The product was centrifuged at 3300 g for 10 minutes to remove unsuspended no-ox-GQDs and the supernatant was collected. The suspension was centrifuged at 16000 g for 1 hour to remove free ssDNA and the precipitate was collected. This purification step was repeated three times until no ssDNA was observed in the supernatant solution by absorption spectroscopy.

### GQD characterization

XPS spectra were collected with a PHI 5600/ESCA system equipped with a monochromatic Al Kα radiation source (hν = 1486.6 eV). High-resolution XPS spectra were deconvoluted with MultiPak software (Physical Electronics) by centering the C-C peak to 284.5 eV, constraining peak centers to ±0.1 eV the peak positions reported in previous literature^36^, constraining full width at half maxima (FWHM) ≤ 1.5 eV, and applying Gaussian-Lorentzian curve fits with the Shirley background. AFM images were collected with an MFP-3D system (Asylum) in tapping mode with an NCL-20 AFM tip (force constant = 48 N/m, Nanoworld). Optical properties of the GQDs were studied with absorbance spectroscopy (UV-3600 Plus, SHIMADZU), photoluminescence spectroscopy (Quantamaster Master 4 L-format, Photon Technologies International), and excitation-emission profiles (Cary Eclipse, Varian). MALDI-TOF mass spectra were acquired on an Autoflex Max (Bruker) with a 355-nm laser, in the positive reflectron mode. Samples were added to CHCA matrix.

### Verification of ssDNA-GQD complexes by AFM

Biotinylated-(GT)_15_-low-ox-GQDs (Bio-(GT)_15_-low-ox-GQDs) were prepared by the mix-and-dry process using 5′-biotinylated-(GT)_15_ and low-ox-GQDs. To form the biotin-streptavidin complex, Bio-(GT)_15_-low-ox-GQDs, containing 10 pmol of biotinylated-(GT)_15_ and 10 pmol of streptavidin were mixed in 0.02 mL DI water. The 50-fold diluted solution was drop cast onto a mica substrate and dried by N_2_ flow. As a negative control, (GT)_15_-low-ox-GQDs were also mixed with streptavidin in the same way. AFM analysis was performed in tapping mode and the height of the GQD complex was determined as the maximum height at the GQD region in the AFM image. Average and standard deviation of the relative portion of structures >1.8 nm were calculated from the height count data of multiple AFM images.

### Molecular dynamics (MD) simulations

MD simulations of ssDNA adsorption on GQDs were performed by NAMD^53,54^ using CHARMM27 and CHARMM36^55^ force field parameters for 100 ns. Obtained trajectories were visualized and analyzed using VMD^56^. Crystallographic data coordinates of A_30_, C_30_, and T_30_ ssDNA as pdb files were generated using 3-D DART software^57^. 5 nm × 5 nm GQDs with sp^2^ hybridized carbon atoms were generated using the VMD plug-in, “nanotube-builder”. Hydroxyl, carbonyl, and carboxyl groups were placed randomly on the GQD surface and edges with VEGA ZZ software^58^. Minimum distance between ssDNA and GQDs was set to 1.4 nm to maintain several ordered water layers that reduced the effects by initial status. The ssDNA and GQDs were then solvated using TIP3P water model^59^ with 150 mM sodium and chloride ions. The water box size was 130 × 80 × 60 Å^3^. Initial ssDNA position and orientation were the same in all simulations.

All computations were performed at constant temperature (300 K) and constant pressure (1 atm). Lennard–Jones potential parameters were set to study the cross-interaction between non-bonded atoms of ssDNA-GQD, GQD-water, and ssDNA-water. All atoms, including hydrogen, were defined explicitly in all simulations. CHARMM force field parameter files were specified to control the interaction between non-bonded atoms of ssDNA-GQD, GQD-water, and ssDNA-water. Exclude parameter was set to scaled1-4, such that all atom pairs directly connected via a linear bond and bonded to a common third atom along with all pairs connected by a set of two bonds were excluded. Electrostatic interactions for the above pairs were modified by the constant factor defined by 1–4scaling, set to 1. Cutoff distance and switching distance function were set to 14 and 10, respectively, and switching parameter set to on, such that the van der Waals energy was smoothly truncated at the cutoff distance starting at the switching distance specified. Pair list distance (pairlistdist) was set to 14 to calculate electrostatic and van der Waals interaction between atoms within a 14 Å radial distance. Integration timestep was set to 2 fs. Each hydrogen atom and the atom to which it was bonded were similarly constrained and water molecules were made rigid. Timesteps between non-bonded evaluation (nonbondedFreq) were set to 1, specifying how often short-range, non-bonded interactions were calculated. Number of timesteps between each full electrostatic evaluation (fullElectFrequency) was set to 2. Number of steps per cycle was set to 10. Langevin dynamics parameter (langevinDamping) was set to 1 to drive each atom in the system to the target temperature. Periodic boundary conditions were specified. Periodic cell center was defined in cellOrigin, to which all coordinates wrapped when wrapAll was set on. Particle Mesh Ewald (PME), applicable only to periodic simulations, was employed as an efficient full electrostatics method that is more accurate and less expensive than larger cutoffs. PME grid dimensions corresponding to the size of the periodic cell were specified. Group-based pressure was used to control the periodic cell fluctuations. Dynamical properties of the barostat and target pressure were controlled by parameters of the Nosé-Hoover Langevin piston. To initiate the simulation, energy minimization for 5000 steps at constant temperature and pressure was performed for all systems that contained ssDNA, GQD, water molecules, and NaCl ions. After minimization, all systems underwent equilibration for 100 ns.

In all MD simulation figures, GQDs are displayed by line representations and black coloring method. ssDNA secondary structures are displayed by New Cartoon representations. Adsorbed residue atoms and oxidation groups on GQDs are displayed by the CPK drawing method with red, green, blue, and magenta coloring for A_30_, C_30_, T_30_, and oxidation groups, respectively.

## Supplementary information

Supplementary information.
