# Supplementary material for: Graphene Quantum Dot Oxidation Governs Noncovalent Biopolymer Adsorption
Source: Sci Rep. 2020 Apr 27;10:7074. doi: 10.1038/s41598-020-63769-z (PMC7184744; doi:10.1038/s41598-020-63769-z)

**Supplementary Information**

**Graphene Quantum Dot Oxidation Governs Noncovalent Biopolymer Adsorption**

*Sanghwa Jeong,^1,†^ Rebecca L. Pinals,^1,†^ Bhushan Dharmadhikari,^2^ Hayong Song,^3^ Ankarao Kalluri,^2^ Debika Debnath,^2^ Qi Wu,^2^ Moon-Ho Ham,^3^ Prabir Patra^2,4^* and Markita P. Landry^1,5,6,7^**

^1^Department of Chemical and Biomolecular Engineering, University of California, Berkeley, Berkeley, CA 94720, USA.

^2^Department of Biomedical Engineering, University of Bridgeport, Bridgeport, CT 06604, USA.

^3^School of Materials Science and Engineering, Gwangju Institute of Science and Technology, Gwangju, 61005, South Korea.

^4^Department of Mechanical Engineering, University of Bridgeport, Bridgeport, CT 06604, USA.

^5^Innovative Genomics Institute (IGI), Berkeley, CA 94720, USA.

^6^California Institute for Quantitative Biosciences, QB3, University of California, Berkeley, Berkeley, CA 94720, USA.

^7^Chan-Zuckerberg Biohub, San Francisco, CA 94158, USA.

†These authors contributed equally

*Corresponding authors, ppatra@bridgeport.edu & [landry@berkeley.edu](mailto:landry@berkeley.edu)


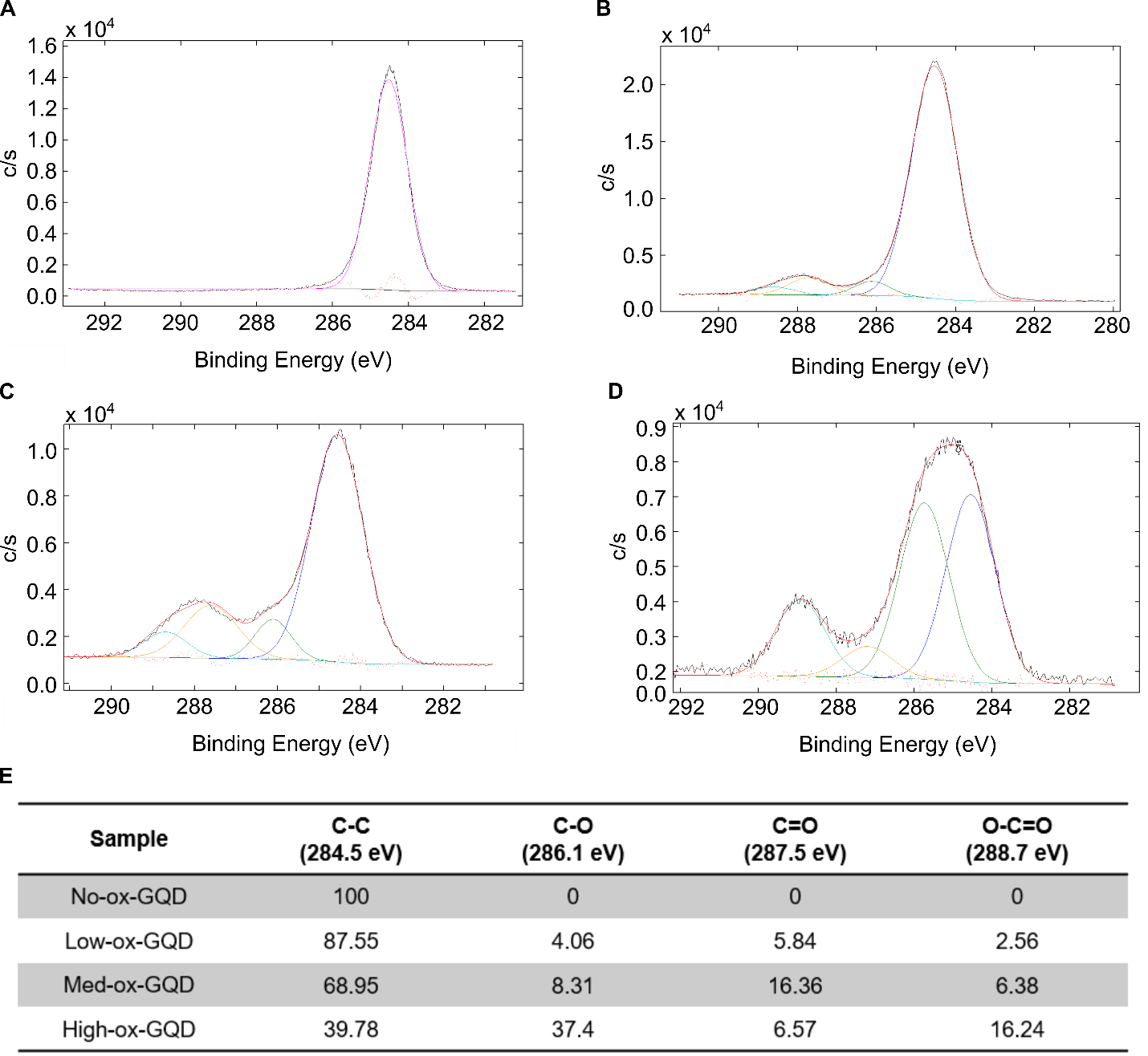


**Figure S1**. **Deconvoluted carbon 1s (C1s) X-ray photoelectron spectroscopy (XPS) characterization of GQDs.** **(A)** no-ox-GQD, **(B)** low-ox-GQD, **(C)** med-ox-GQD, and **(D)** high-ox-GQD samples included in Fig. 1B. **(E)** Relative peak areas for C1s chemical bonds in each sample.


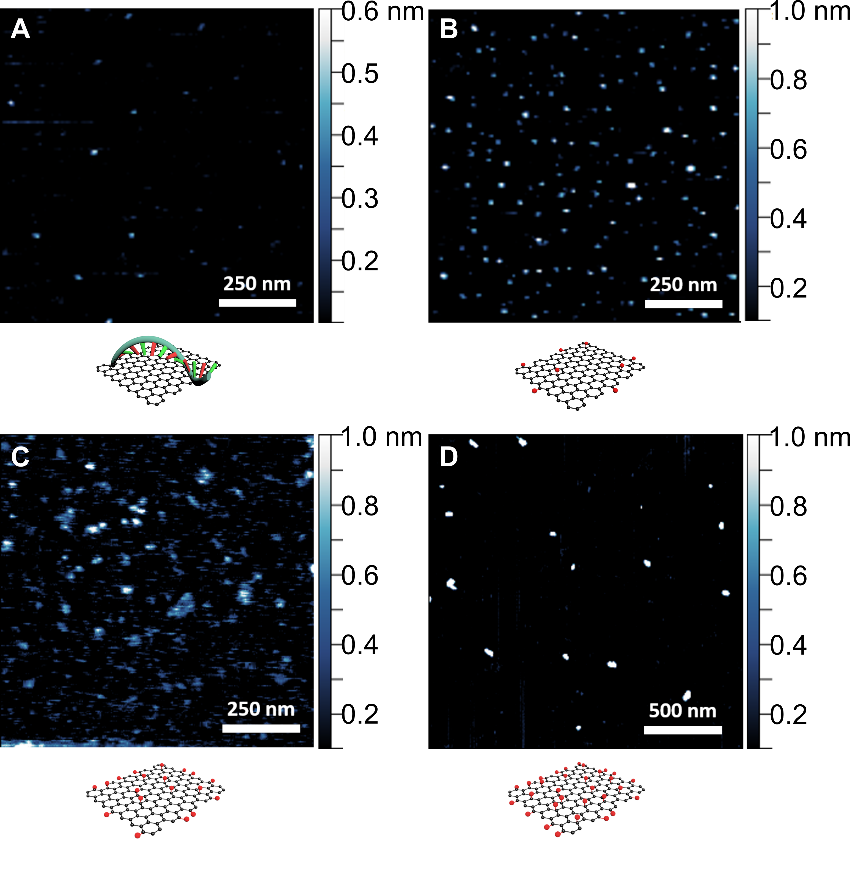


**Figure S2**. **AFM characterization of GQDs.** AFM images and accompanying schematics for **(A)** (GT)_15_-no-ox-GQDs, **(B)** low-ox-GQDs, **(C)** med-ox-GQDs, and **(D)** high-ox-GQDs. Average height of each type of GQD is 0.35 ± 0.10, 0.83 ± 0.28, 0.82 ± 0.75, and 2.0 ± 0.91 nm for (GT)_15_-no-ox-GQDs, low-ox-GQDs, med-ox-GQDs, and high-ox-GQDs, respectively.


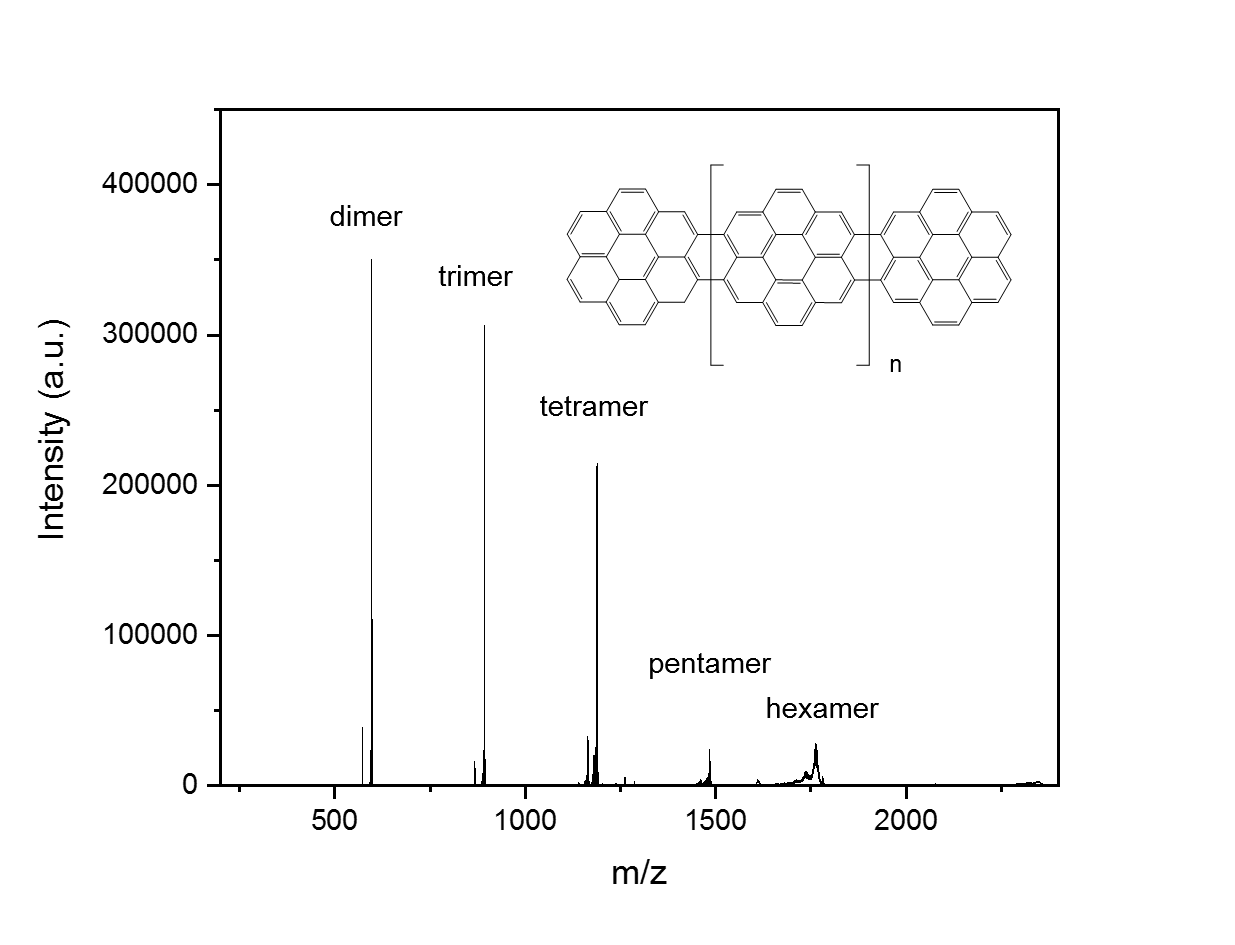


**Figure S3**. **Matrix-assisted laser desorption/ionization time-of-flight mass spectroscopy (MALDI-TOF MS) characterization of no-ox-GQDs**. Peak bands observed at m/z = 596, 892, 1188, 1484, and 1780 are attributed to planar coronene dimer, trimer, tetramer, and pentamer, and hexamer structures, respectively.

**
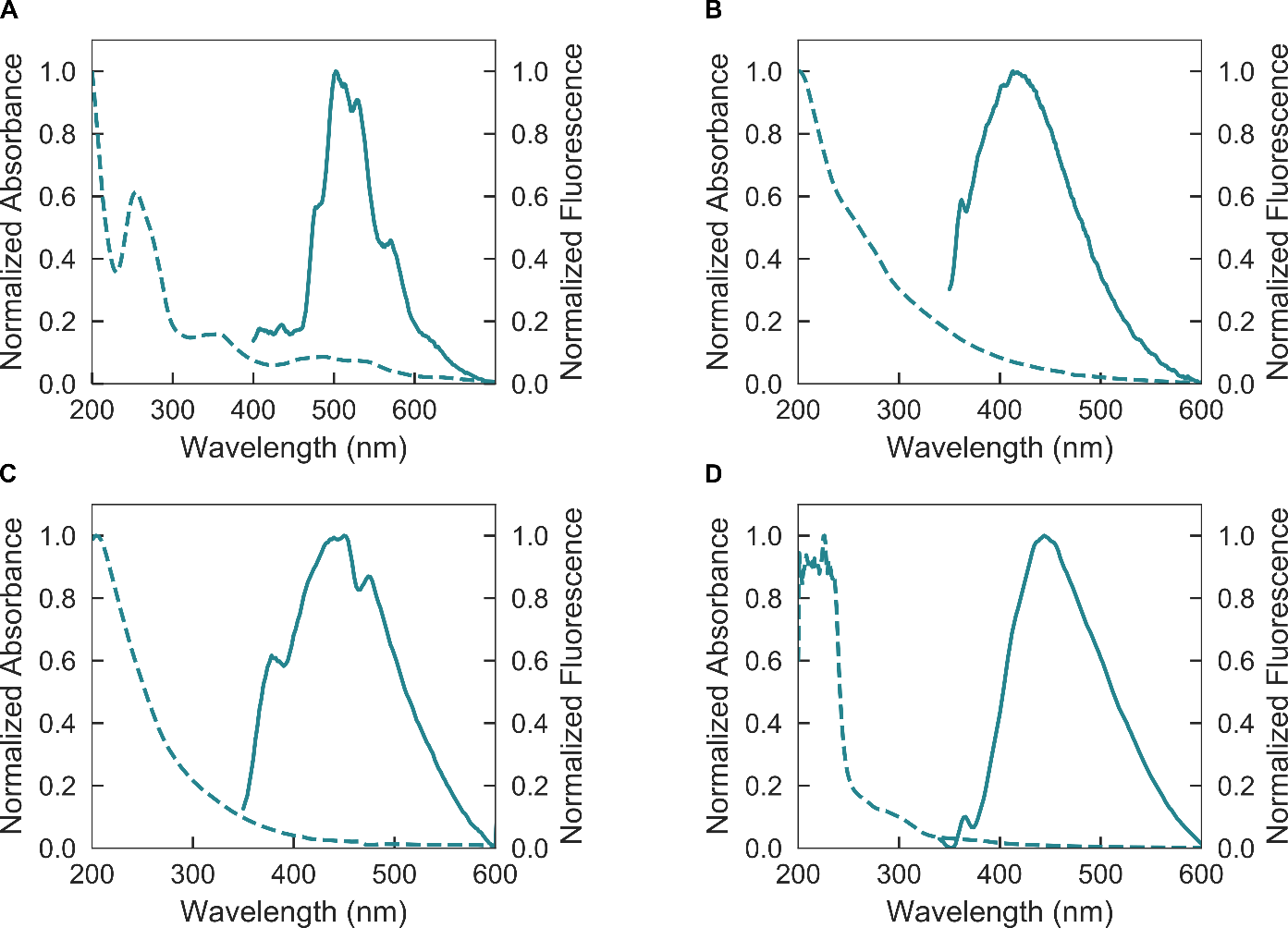
**

**Figure S4**. **Optical absorption and emission characterization of GQDs.** Normalized absorption (dashed) and fluorescence emission (solid) spectra of **(A)** (GT)_15_-no-ox-GQDs (excitation 340 nm), **(B)** low-ox-GQDs (excitation 320 nm), **(C)** med-ox-GQDs (excitation 320 nm), and **(D)** high-ox-GQDs (excitation 340 nm) in water.


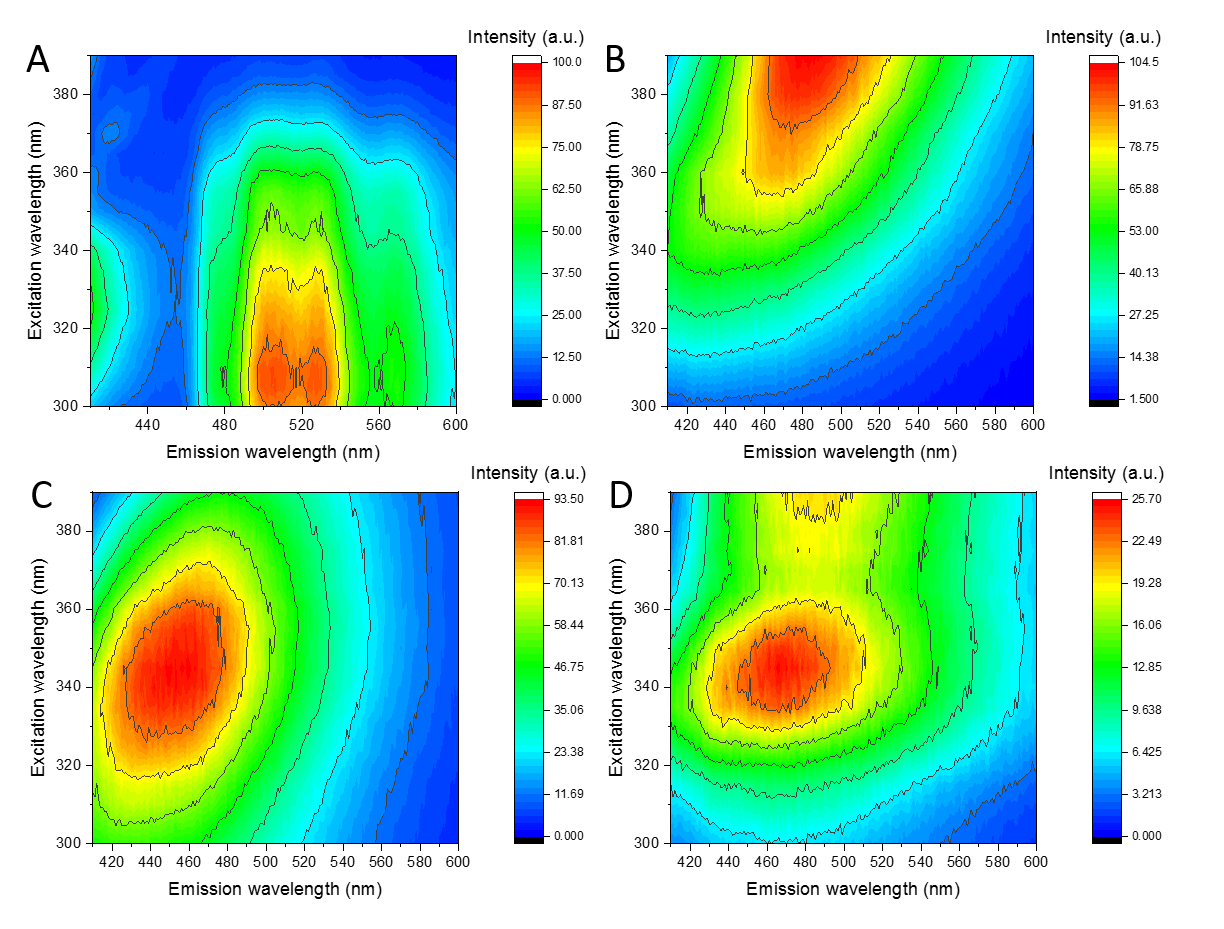


**Figure S5. Excitation-emission profiles of GQDs.** **(A)** (GT)_15_-no-ox-GQDs, **(B)** low-ox-GQDs, **(C)** med-ox-GQDs, and **(D)** high-ox-GQDs in water. All spectra were collected in intervals of 5 nm for excitation wavelength.


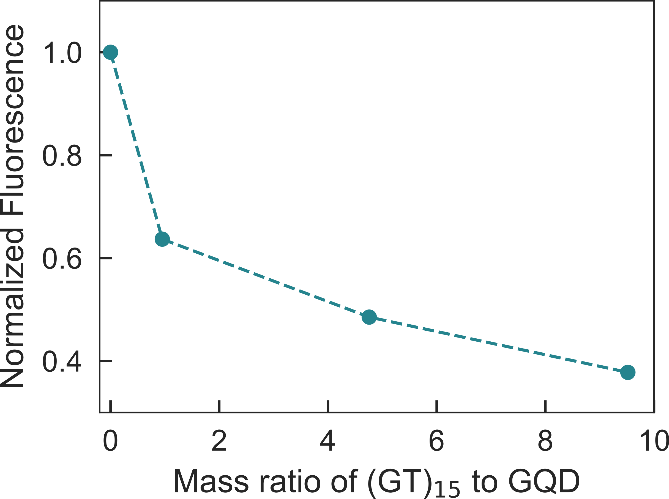


**Figure S6. Fluorescence intensity of (GT)_15_-low-ox-GQDs depends on the mass ratio of (GT)_15_ to low-ox-GQD.** Integrated, normalized fluorescence intensities upon (GT)_15_ ssDNA adsorption to low-ox-GQDs, with curve fits added to guide the eye.


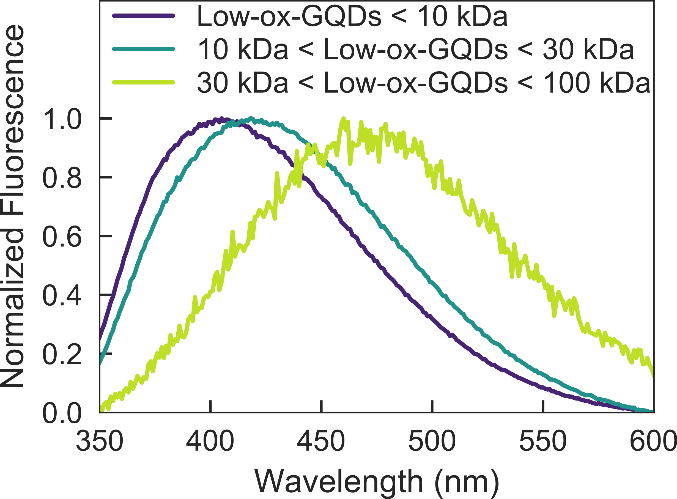


**Figure S7. Low-ox-GQD fluorescence as a function of GQD size.** Normalized fluorescence spectra demonstrate that larger low-ox-GQDs exhibit redshifted peak emission in comparison to smaller low-ox-GQDs. GQDs were size sorted by filtering with varying molecular weight cutoff (MWCO) centrifugal filters (Amicon Ultra-15, Ultracel, Millipore; 10 kDa, 30 kDa, and 100 kDa MWCO) at 3220 g for 30 min.


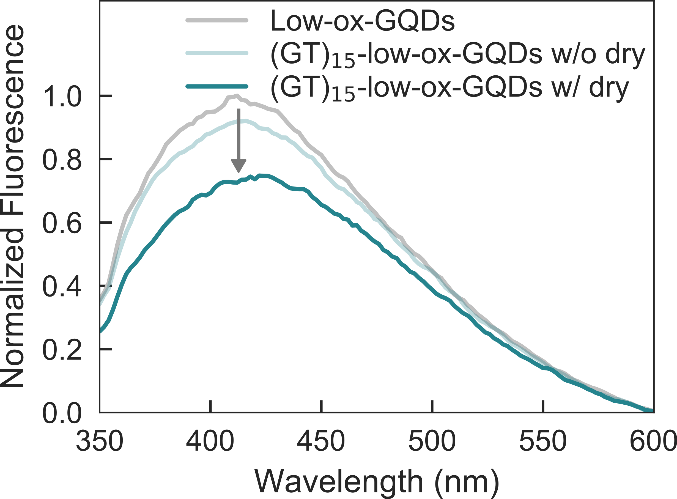


**Figure S8**. **Effectiveness of vacuum drying for ssDNA adsorption to low-ox-GQDs.** Normalized fluorescence emission spectra of low-ox-GQDs after vacuum evaporation (gray), (GT)_15_ and low-ox-GQD mixture without vacuum evaporation (light blue), and (GT)_15_ and low-ox-GQD mixture with vacuum evaporation (dark blue). Concentrations of all low-ox-GQD samples are equivalent.


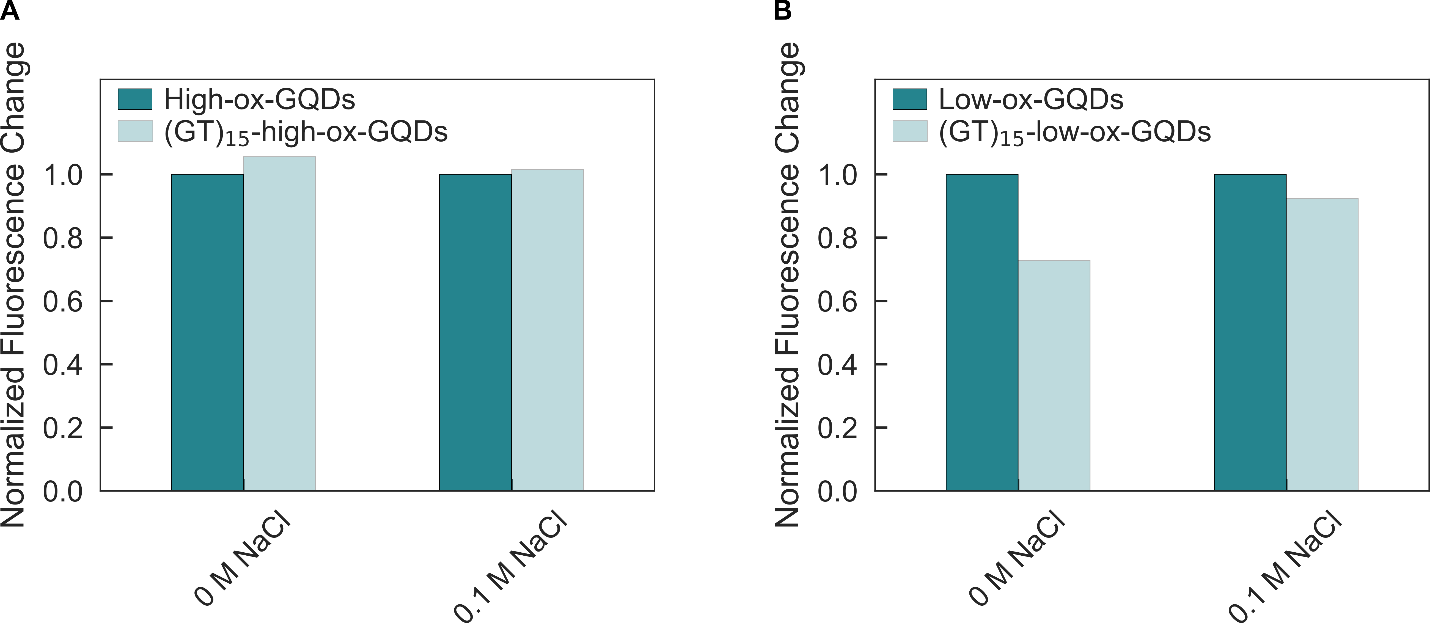


**Figure S9. Impact of ionic strength on ssDNA adsorption to GQDs.** Normalized fluorescence change of **(A)** (GT)_15_-high-ox-GQDs and **(B)** (GT)_15_-low-ox-GQDs in water vs. in 100 mM NaCl, demonstrating the lack of stability of ssDNA-coated low-ox-GQDs in higher salt conditions.


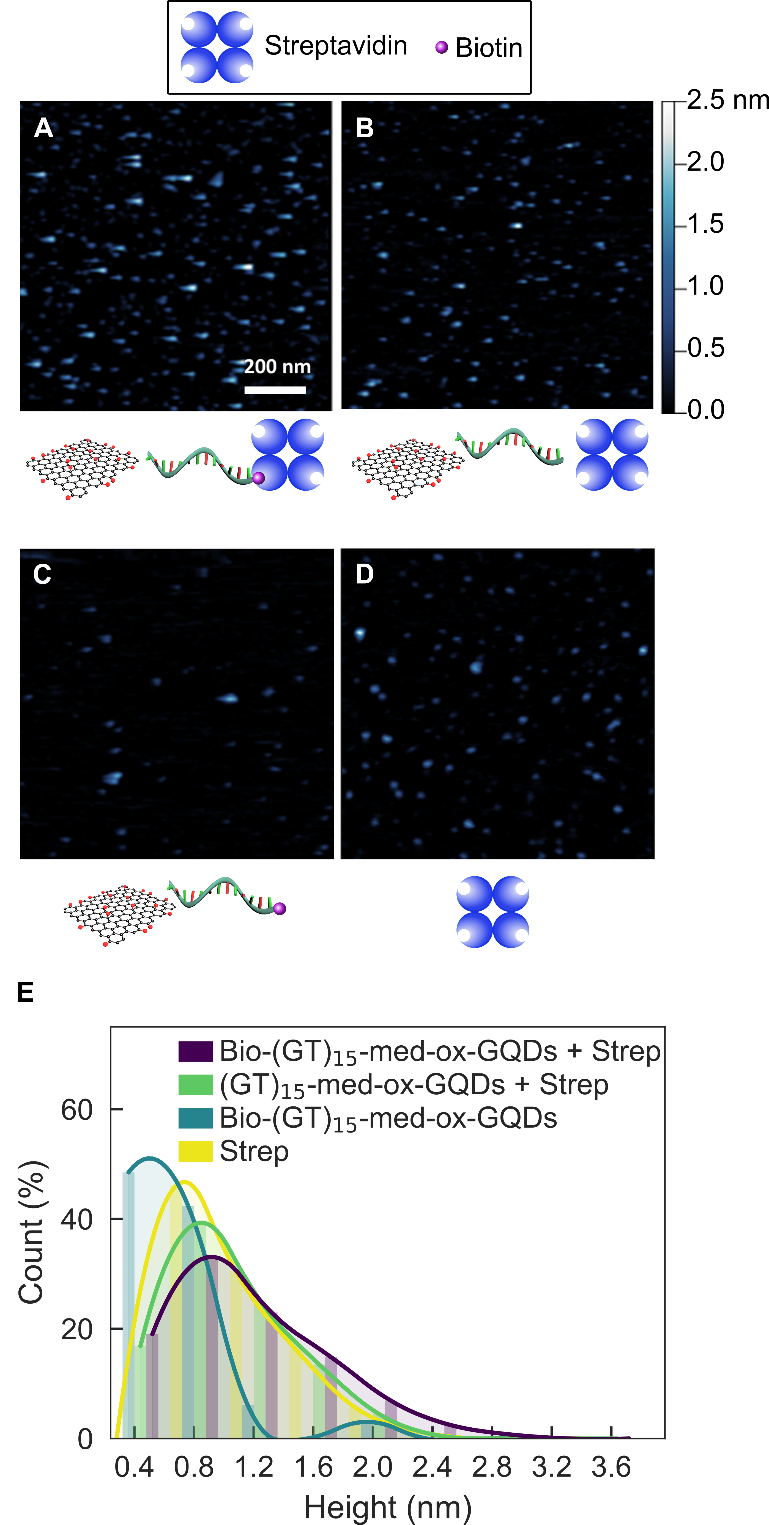


**Figure S10. AFM verification of no ssDNA adsorption on med-ox-GQDs.** AFM images and accompanying schematics for **(A)** biotinylated-(GT)_15_-med-ox-GQDs and streptavidin (Bio-(GT)_15_-med-ox-GQD + Strep), **(B)** (GT)_15_-med-ox-GQDs and streptavidin ((GT)_15_-med-ox-GQD + Strep), **(C)** biotinylated-(GT)_15_-med-ox-GQDs (Bio-(GT)_15_-med-ox-GQD), and **(D)** streptavidin (Strep). **(E)** Corresponding height distribution histograms. Bin width is 0.4 nm and curve fits are added to guide the eye.


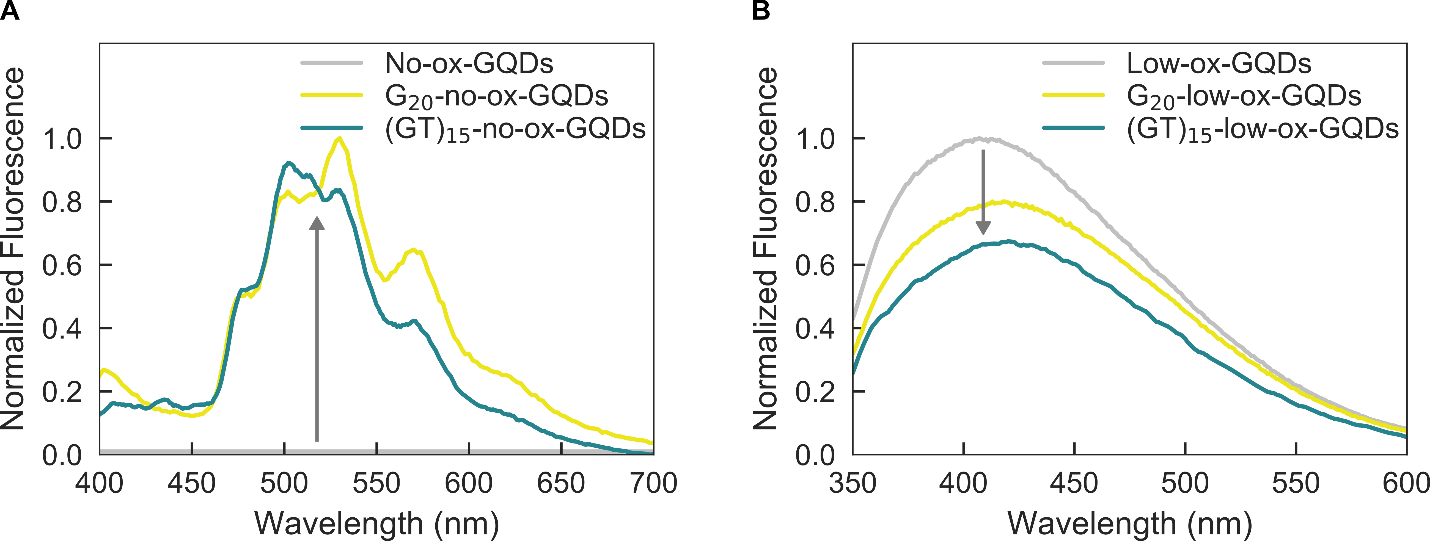


**Figure S11.** **Normalized fluorescence emission spectra to probe ssDNA sequence dependence for adsorption to GQDs.** Fluorescence of G_20_-GQDs (yellow) compared to (GT)_15_-GQDs (blue) for **(A)** no-ox-GQDs and **(B)** low-ox-GQDs. All GQD fluorescence spectra are normalized by the absorbance at 320 nm.

**
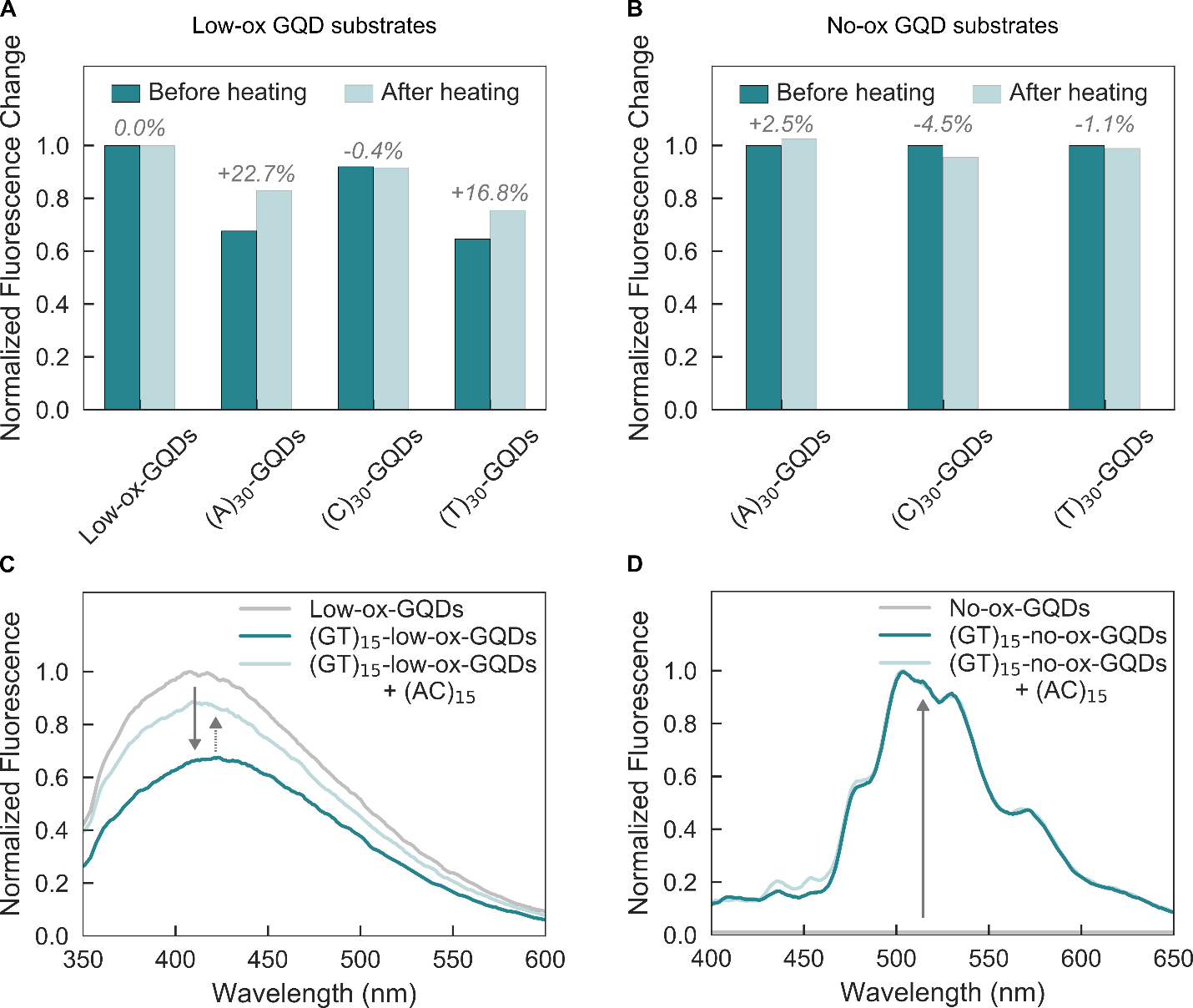
**

**Figure S12**. **ssDNA desorption from low- and no-ox-GQDs indicates strength of noncovalent binding interactions is inversely proportional to GQD oxidation level.** Fluorescence intensity change of **(A)** ssDNA-low-ox-GQDs and **(B)** ssDNA-no-ox-GQDs induced by thermal desorption of ssDNA after heating ssDNA-GQD samples (in water) at 50 ℃ for 2 hours. Normalized fluorescence emission spectra of **(C)** (GT)_15_-low-ox-GQDs and **(D)** (GT)_15_-no-ox-GQDs, after adding five-fold excess of complementary ssDNA, (AC)_15_ (in water). All GQD fluorescence spectra are normalized by the absorbance at 320 nm.


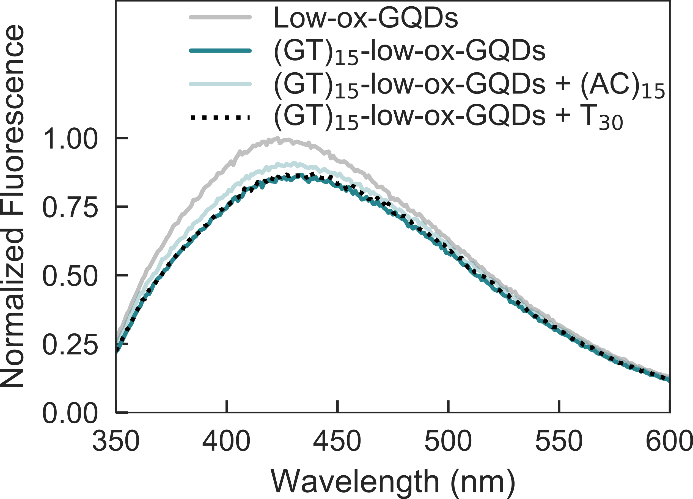


**Figure S13. Effect of non-complementary ssDNA addition to (GT)_15_-low-ox-GQD.** Normalized fluorescence spectra of (GT)_15_-low-ox-GQD in water after the addition of complementary (AC)_15_ and T_30_ ssDNA.


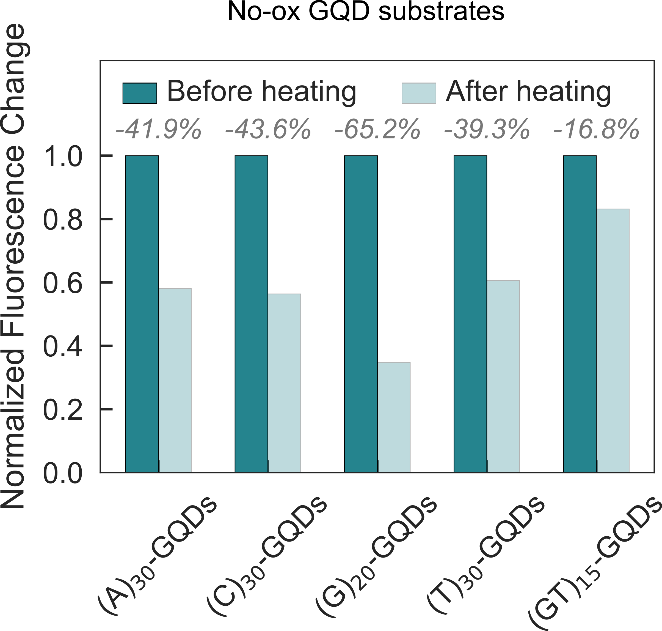


**Figure S14. Thermally-induced ssDNA desorption from ssDNA-no-ox-GQDs.** Fluorescence intensity change of ssDNA-no-ox-GQDs induced by thermal desorption of ssDNA after heating the mixture at 95 ℃ for 2 hours.

**
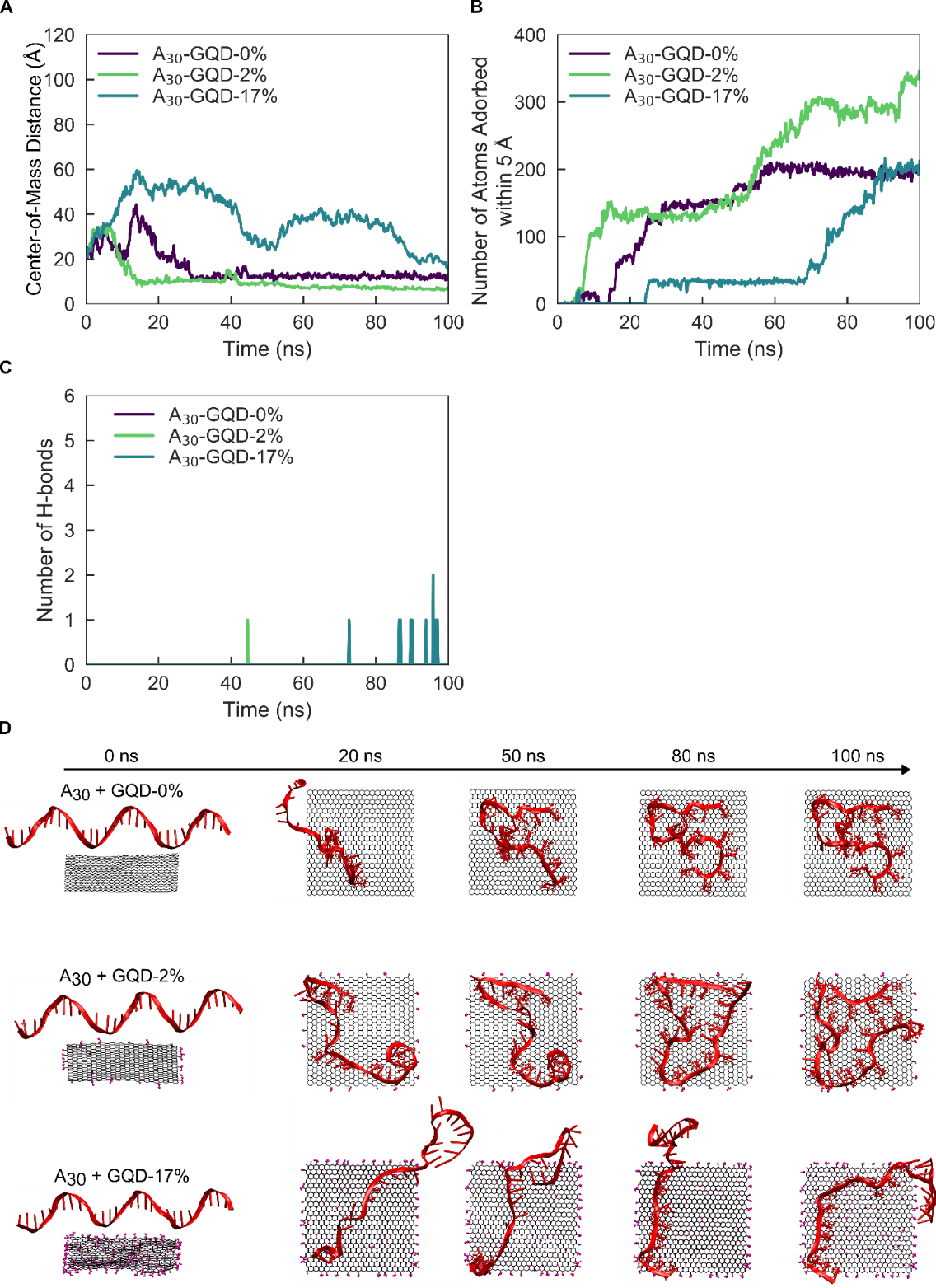
**

**Figure S15. Molecular dynamics simulations of A_30_ ssDNA adsorbing to GQDs of varying oxidation levels.** Time-dependent **(A)** center-of-mass distance, **(B)** number of atoms adsorbed within 5 Å of the GQD surface, and **(C)** number of hydrogen bonds for A_30_ ssDNA adsorbing to GQD-0%, GQD-2%, and GQD-17%. **(D)** Initial (left) and final (right) configurations of A_30_ ssDNA with GQD-0%, GQD-2%, and GQD-17% for a 100 ns simulation.


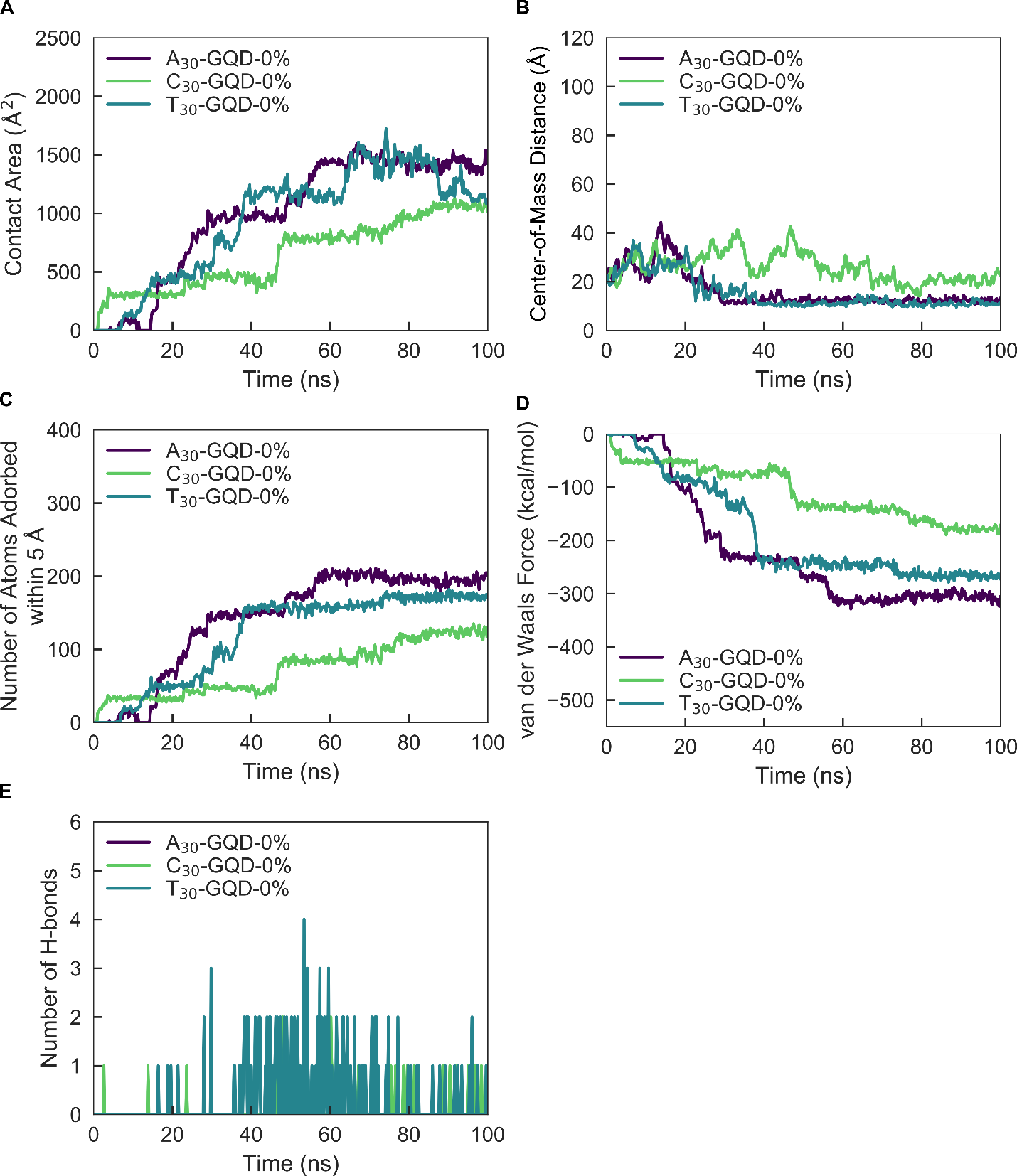


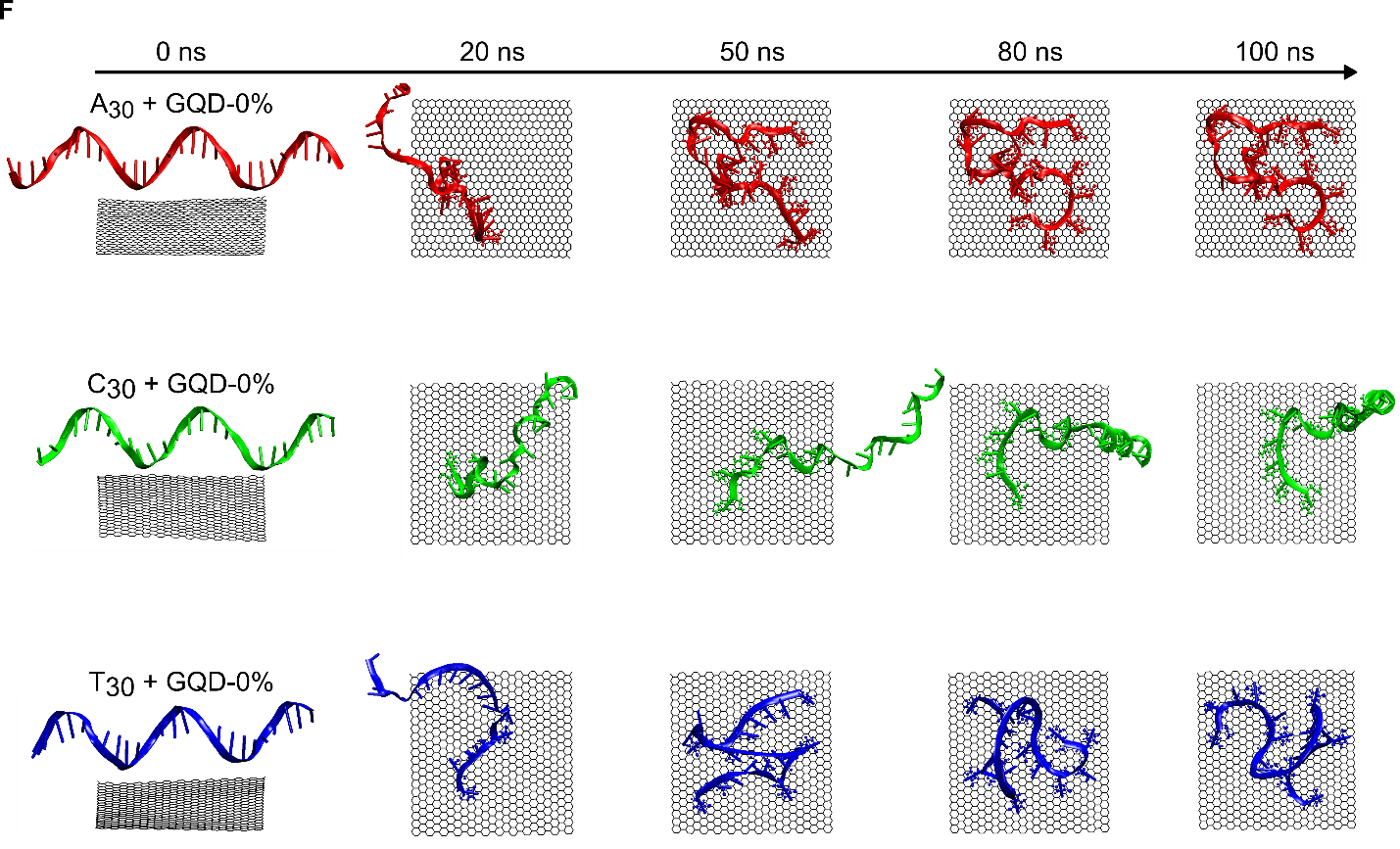


**Figure S16. Molecular dynamics simulations of A_30_, C_30_, and T_30_ ssDNA adsorbing to GQDs without oxidation, GQD-0%.** Time-dependent **(A)** contact area, **(B)** center-of-mass distance, **(C)** number of atoms adsorbed within 5 Å of the GQD surface, **(D)** van der Waals interactions, and **(E)** number of hydrogen bonds for A_30_, C_30_, and T_30_ ssDNA adsorbing to GQD-0%. **(F)** Initial (left) and final (right) configurations of ssDNA with GQD-0% for a 100 ns simulation.


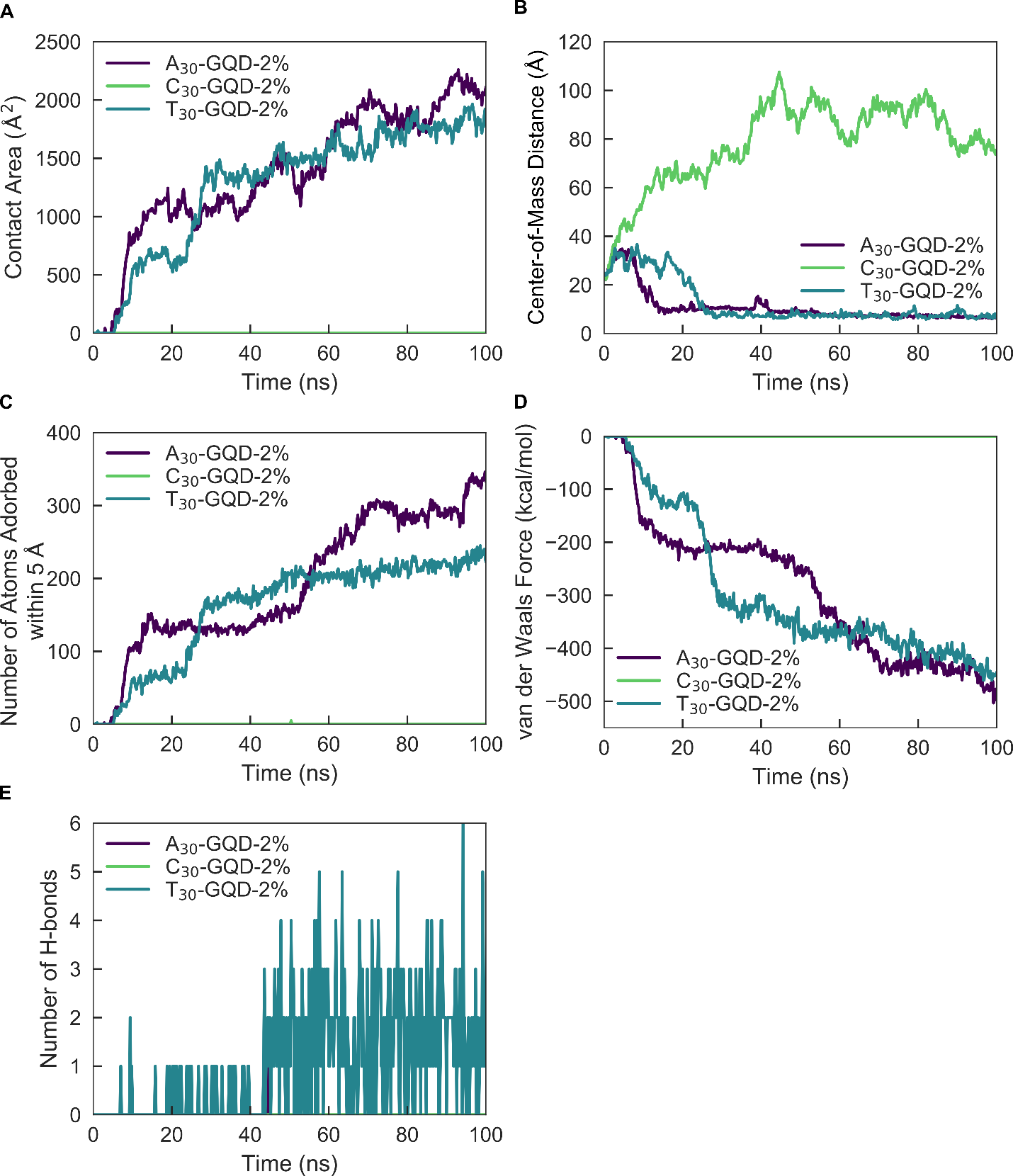


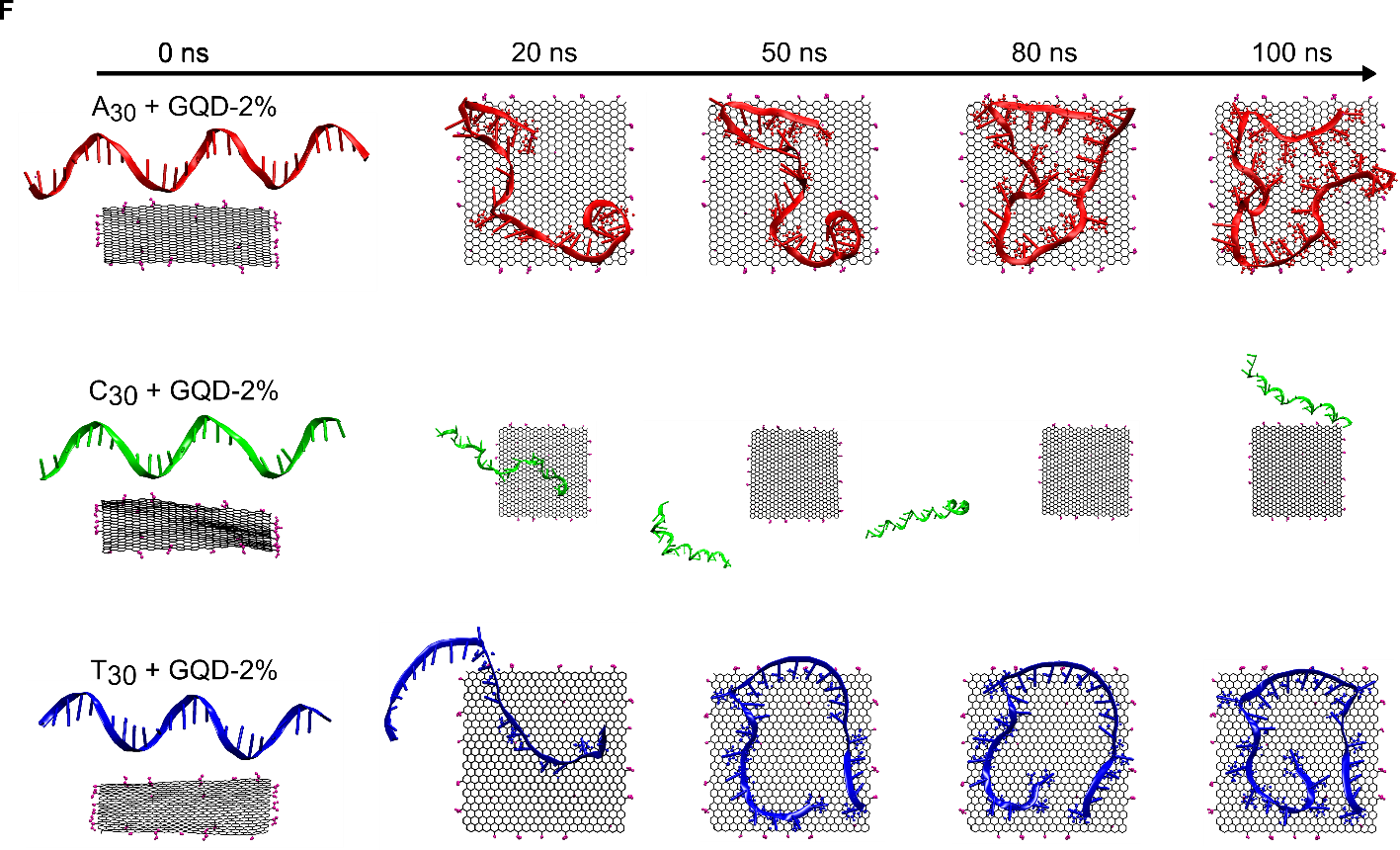


**Figure S17. Molecular dynamics simulations of A_30_, C_30_, and T_30_ ssDNA adsorbing to GQDs low oxidation, GQD-2%.** Time-dependent **(A)** contact area, **(B)** center-of-mass distance, **(C)** number of atoms adsorbed within 5 Å of the GQD surface, **(D)** van der Waals interactions, and **(E)** number of hydrogen bonds for A_30_, C_30_, and T_30_ ssDNA adsorbing to GQD-2%. **(F)** Initial (left) and final (right) configurations of ssDNA with GQD-2% for a 100 ns simulation. Note zoomed-out view of C­_30_ due to larger distance of ssDNA to GQD surface; GQD size is the same throughout.

**Table S1. Biomolecule adsorption on GQDs is dependent on GQD oxidation level.**


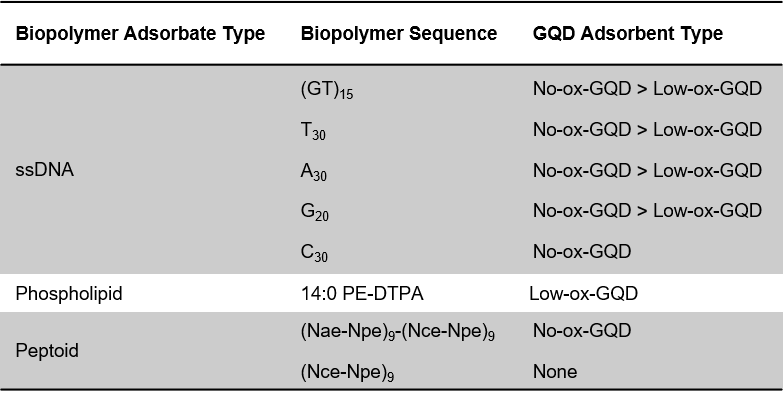

Supplement: Supplementary file 1 — Supplementary information. [file 41598_2020_63769_MOESM1_ESM.docx]
